# Applications and Action Mechanisms of *Yucca schidigera* Extract in Livestock, Poultry and Aquatic Animals

**DOI:** 10.3390/ani16152376

**Published:** 2026-08-03

**Authors:** Shiqi Xue, Qiwei Liu, Jingrong Feng, Yang Feng, Huijin Jia, Lanlan Yi, Sumei Zhao, Junhong Zhu

**Affiliations:** Yunnan Provincial Key Laboratory of Animal Nutrition and Feed, College of Animal Science and Technology, Yunnan Agricultural University, Kunming 650201, China; 2024210423@stu.ynau.edu.cn (S.X.); 18108817132@163.com (Q.L.); 18736030982@163.com (J.F.); 18336675074@163.com (Y.F.); 2025110121@stu.ynau.edu.cn (H.J.); yilanlan0217@163.com (L.Y.)

**Keywords:** *Yucca schidigera* extract, livestock and poultry production, bioactive components, gut health, enteric methane mitigation, phytogenic feed additives, animal nutrition, antioxidants, sustainable livestock production

## Abstract

*Yucca schidigera* extract (YSE) is a plant-derived feed additive rich in steroidal saponins, polyphenols, and polysaccharides, with demonstrated anti-inflammatory, antioxidant, antibacterial, and ammonia-binding properties. This review systematically synthesizes the major bioactive components, core mechanisms of action, including modulation of the Nrf2/ARE and NF-κB pathways, regulation of ruminal fermentation and ammonia-nitrogen metabolism, and maintenance of intestinal barrier integrity, and the species-specific application effects of YSE in pigs, poultry, ruminants, and aquatic animals. Most studies have shown that YSE can improve growth performance, reproductive efficiency, and product quality while concurrently reducing ammonia and methane emissions. However, the magnitude and consistency of these benefits vary considerably across species, dosage, and experimental conditions, with most evidence derived from controlled trials rather than commercial-scale validation. By integrating cross-species dose–response data and identifying critical knowledge gaps, including product standardization, long-term safety, and economic feasibility under commercial conditions, this review provides a theoretical and practical foundation for the evidence-based application of YSE as a sustainable feed additive. In the context of global efforts to reduce antibiotic use and mitigate environmental pollution from livestock production, YSE represents a promising, multifunctional tool for advancing ecological and low-carbon animal husbandry.

## Simple Summary

Against the backdrop of continuous intensification of global livestock production and the progressive enforcement of regulatory policies restricting antibiotic application in animal feed globally, the development of safe and environmentally friendly antibiotic alternatives has become an urgent research priority. As a phytogenic feed additive, *Yucca schidigera* extract (YSE) is abundant in saponins, polyphenols and polysaccharides, and exhibits multiple biological activities including anti-inflammatory, antioxidant, ammonia-lowering and gut microbiota-modulating effects, which have drawn extensive scholarly attention. Based on a systematic collation of existing relevant research, this paper comprehensively summarizes the main bioactive components, core biological functions, underlying mechanisms of action, and current applications of YSE in livestock, poultry and aquatic animal production. This study further discusses the limitations of current research and proposes prospects for future investigation, aiming to clarify the application potential of YSE in the livestock farming industry.

## 1. Introduction

With the continuous intensification of global livestock production, antibiotic resistance, food safety, and farming-derived environmental pollution have become increasingly prominent [1], driving the search for safe, green antibiotic alternatives, with plant extracts attracting wide interest due to their natural, low-residue, and multifunctional properties [2]. *Yucca schidigera*, a perennial Asparagaceae plant native to arid regions of the southwestern US and northwestern Mexico [3,4], has stems, bark, and roots rich in bioactive constituents [5], serving as the main source for commercial extracts. Preparation methods vary depending on plant part and product type: conventional industrial extraction employs mechanical crushing, cold pressing, evaporation to liquid, or spray-drying to powder [6,7]; laboratory and industrial saponin extraction typically uses aqueous methanol or ethanol, followed by n-butanol partition and chromatography [8]. Recent green technologies include ultrasound-assisted extraction (UAE) and deep eutectic solvents (DES), with UAE showing superior saponin content and antioxidant activity over DES [6]. Additionally, Falev et al. [9] used 2D NMR and LC-MS for phytochemical comparison of four Yucca species, providing new tools for species identification and quality control.

*Yucca schidigera* extract (YSE) is a complex mixture, with steroidal saponins as its primary bioactive components, which can reach up to 28.1% as determined by spectrophotometric methods [10]. In addition to saponins, yucca bark and roots are rich in polyphenolic compounds, including trans-resveratrol, yuccaols A–E featuring unique spirocyclic structures, and 3,3′,5,5′-tetrahydroxy-4′-methoxystilbene [11]. These structurally diverse active constituents confer multiple biological activities to YSE, such as anti-inflammatory, antioxidant, antibacterial, anti-tumor, and glucose- and lipid-metabolism-modulating effects [4,12], thereby rendering it widely applied in the food, cosmetic, pharmaceutical, livestock and poultry production sectors. Numerous studies have confirmed that YSE not only improves the growth and productive performance of livestock and poultry, but also reduces odorous gas emissions at the source from farm operations, thus contributing to the advancement of ecological and healthy husbandry models [13,14].

Although several reviews have addressed the general functions of YSE [15,16,17,18], none has provided a comprehensive integration of its molecular mechanisms with cross-species efficacy data. For instance, Zúñiga-Serrano et al. [19] focused primarily on antimicrobial and digestive effects, without systematically dissecting the signaling pathways (e.g., Nrf2/ARE, NF-κB, MAPK) or the distinct contributions of individual bioactive fractions (saponins, polyphenols, and polysaccharides); Wang et al. [2] offered a broad overview of phytogenic feed additives but treated YSE as one of many examples, without in-depth coverage of its species-specific dose–response characteristics; and Ghallab et al. [20] provided an updated phytochemical profile oriented toward human health rather than animal production. Moreover, in livestock and poultry production, the efficacy of YSE varies considerably across animal species, physiological stages, and supplemental dosages [21,22,23], yet existing studies are mostly confined to single-animal models, lacking systematic cross-species comparisons and mechanistic integration. Given the substantial body of new evidence published since these earlier reviews and the growing practical need for evidence-based application guidelines, a dedicated synthesis is timely and warranted. Therefore, the present review offers a systematic, mechanism-based synthesis of the major bioactive components, core functionalities, and action mechanisms of YSE, alongside a comprehensive cross-species comparison across swine, poultry, ruminants, and aquatic animals, with the aim of providing a theoretical and practical basis for the scientific utilization of YSE as a phytogenic feed additive in contemporary animal production.

## 2. Review Methodology

This narrative review aims to integrate and critically analyze the relevant scientific literature on the efficacy of YSE in livestock, poultry, and aquatic animals production. Relevant literature was mainly retrieved from Web of Science, China National Knowledge Infrastructure (CNKI), and PubMed. The search keywords include: “*Yucca schidigera*,” “*Yucca schidigera* extract,” “YSE,” “Yucca saponin,” “Yucca and pig,” “Yucca and ruminant,” “Yucca and cattle,” “Yucca and sheep,” “Yucca and poultry,” and “Yucca and fish,” “Yucca and shrimp” covering publications from 2017 to 2026.

Initial search returned approximately 400 records. Explicit inclusion and exclusion criteria were established: only English-language articles focusing on the primary bioactive components, biological functions, and applications of *Yucca schidigera* extract in animal production were included, whereas conference abstracts, non-English publications, and irrelevant studies were excluded. Following a two-stage screening process, which involved initial assessment by titles and abstracts followed by full-text evaluation, a total of 145 articles were ultimately retained for integrative narrative synthesis in this review. Since the present study solely utilizes data and materials from previously published literature, ethical approval is not required for this research.

## 3. Main Bioactive Components

### 3.1. Steroidal Saponins

Steroidal saponins are the most representative bioactive components of YSE and constitute the primary material basis for its biological effects. The steroidal saponins in yucca mainly include sarsasapogenin, smilagenin, hecogenin, and their glycoside conjugates [24]. Chemically, steroidal saponins consist of a steroid or triterpenoid skeleton linked to a glycan chain via glycosidic bonds, and are typical amphiphilic molecules possessing both lipophilic and hydrophilic properties [25,26]. The core biological properties of steroidal saponins derive from their surface activity. Saponin molecules can form foams in aqueous solutions and reduce liquid surface tension. This detergent-like property enables them to bind to cholesterol in cell membranes, thereby altering membrane permeability and fluidity [27,28]. Consequently, saponins exhibit antithrombotic activity, can serve as vaccine adjuvants, enhance drug cytotoxicity against cancer cells, and possess potential therapeutic value [29]. Furthermore, yucca saponins share structural similarity with glucocorticoids secreted during animal stress, allowing them to competitively bind to glucocorticoid receptors and exert anti-stress effects [30]. Studies have also shown that yucca saponins possess antiparasitic effects, mediated through the formation of complexes with cholesterol in protozoan cell membranes, leading to disruption of membrane integrity and subsequent cell lysis [20,31].

### 3.2. Yucca Polyphenols

Yucca polyphenols mainly include stilbene derivatives such as resveratrol and yuccaols A–E, as well as flavonoids, and their physiological functions are primarily manifested in three aspects: antioxidant, anti-inflammatory, and antibacterial activities [19,32]. With respect to antioxidant effects, polyphenols can directly scavenge free radicals (superoxide anion and hydroxyl radical), inhibit lipid peroxidation, and activate the nuclear factor erythroid 2-related factor 2 (Nrf2) signaling pathway, thereby upregulating the expression of endogenous antioxidant enzymes, including superoxide dismutase (SOD), glutathione peroxidase (GSH-Px), and catalase (CAT) [33]. In terms of anti-inflammatory activity, yucca polyphenols suppress the expression of cyclooxygenase-1 (COX-1) and cyclooxygenase-2 (COX-2) in arachidonic acid metabolism, reducing the production of pro-inflammatory mediators such as thromboxane and prostaglandin E_2_ (PGE_2_). Moreover, by inhibiting the activation of the nuclear factor-kappa B (NF-κB) and mitogen-activated protein kinase (MAPK) signaling pathways, they decrease the production of inflammatory cytokines, including Tumor Necrosis Factor-α (TNF-α), Interleukin-6 (IL-6), and COX-2, thereby exerting anti-inflammatory effects [33,34]. Resveratrol, a naturally potent antioxidant polyphenolic compound, plays important roles in medical, food, and animal production fields [35,36]. Studies have demonstrated that dietary supplementation of resveratrol in ewes promotes the proliferation of certain cellulolytic bacteria, while inhibiting protozoa and methanogens, thus reducing methane emissions [37]. Furthermore, both resveratrol and saponins have been shown to inhibit urease activity, which can reduce ammonia production from urea decomposition at the source, and they also decrease ammonia emissions through physicochemical adsorption and by significantly reducing the abundance of ammonifying bacteria [22,38].

### 3.3. Yucca Polysaccharides

Yucca polysaccharides represent another important class of bioactive components in YSE. Fan et al. [39] isolated and purified a polysaccharide designated *Yucca schidigera* polysaccharide B (YPB), which is a heteropolysaccharide composed of monosaccharides including rhamnose (Rha), arabinofuranose (Araf), galactopyranose (Galp), and glucopyranose (Glcp) linked via glycosidic bonds. Yucca polysaccharides possess relatively high molecular weights, complex spatial conformations, and abundant reactive groups. The most prominent function of yucca polysaccharides is their potent adsorption and binding capacity for harmful gases such as ammonia. Their molecular structure contains numerous polar groups, including hydroxyl and carboxyl moieties, which can interact with ammonia molecules or ammonium ions through hydrogen bonding, van der Waals forces, and electrostatic interactions, forming stable complexes and thereby reducing ammonia release in the intestinal tract and within the housing environment [38,40]. Furthermore, yucca polysaccharides can act as prebiotics, selectively promoting the proliferation of beneficial intestinal bacteria (*Bifidobacterium* and *Lactobacillus*) and maintaining gut microecological balance. Some studies have also indicated that yucca polysaccharides possess immunomodulatory activity, activating macrophages, promoting cytokine secretion, and enhancing non-specific immune function in animals [41]. The main active ingredients, biological functions and action sites of YSE are shown in Table 1.

## 4. Core Functions and Mechanisms of Action

### 4.1. Anti-Inflammatory and Antioxidant Effects

In animals, the Kelch-like ECH-associated protein 1 (Keap1)/Nrf2 signaling pathway is a critical mechanism for defending against oxidative stress-induced damage. Under oxidative stress, the Keap1-Nrf2 complex dissociates, followed by the nuclear translocation of Nrf2 and its subsequent binding to the antioxidant response element (ARE). This mechanism has been well validated across in vivo studies of livestock and poultry, mammalian cell lines, and rodent models [47,48,49,50]. The Nrf2/ARE signaling pathway serves as the central regulatory hub of the endogenous antioxidant system [51]. Fan et al. [39] conducted experiments using the IPEC-J2 porcine jejunal epithelial cell line, and confirmed that *Yucca schidigera* polysaccharide activates the Nrf2 signaling pathway by binding to the mannose receptor (MR) and epidermal growth factor receptor (EGFR). This conclusion has been validated by receptor-specific inhibitor assays. Activation of the Nrf2 pathway promotes the expression of downstream antioxidant enzymes, including SOD, GSH-Px, CAT, and heme oxygenase-1 (HO-1), thereby scavenging reactive oxygen species (ROS) and alleviating oxidative stress-induced injury [39,51,52,53]. The synergistic action of these enzymes constitutes a complete intracellular antioxidant defense network.

The MAPK pathway comprises three major subpathways, namely extracellular signal-regulated kinase 1 and 2 (ERK1/2), c-Jun N-terminal kinase (JNK), and p38, which play pivotal roles in cell proliferation, differentiation, and inflammatory responses [54,55]. The modulation of MAPK phosphorylation by Yucca-derived phytochemicals, as evidenced in cell-based studies (e.g., RAW 264.7 macrophages), contributes to the regulation of inflammatory cytokine production and apoptosis [33]. In a carrageenan-induced rat paw edema model, Yucca gigantea extract significantly reduced TNF-α and COX-2 levels, improved histological skin architecture, and restored GSH levels [56].

NF-κB is a well-characterized transcription factor. Studies in mammalian cell lines have confirmed that this factor modulates inflammatory responses by promoting the expression of pro-inflammatory cytokines [57,58]. Research has shown that under ammonia stress, YSE downregulates the messenger ribonucleic acid (mRNA) expression levels of TNF-α, IL-8 and IL-1β in common carp [49], which suggests that the NF-κB signaling pathway may be involved in this regulatory process. Studies conducted in mouse intestinal epithelial cells have demonstrated that resveratrol alleviates inflammation by reducing the activity of the Janus kinase 2 (JAK2)/signal transducer and activator of transcription 3 (STAT3) and the O-linked N-acetylglucosamine (O-GlcNAc) modification (O-GlcNAcylation) of STAT3 [59]. And the JAK/STAT signaling pathway serves as a core pathway for signal transduction of multiple cytokines and growth factors [60,61]. As summarized in reviews, *Yucca* spp. reduces the release of pro-inflammatory cytokines by inhibiting the activation of the MAPK, NF-κB and JAK/STAT pathways [56,62], thereby exerting anti-inflammatory effects and maintaining immune homeostasis [33,43].

### 4.2. Modulation of Ruminal and Ammonia-Nitrogen Metabolism

Yucca saponins exhibit pronounced antiprotozoal properties, which represent one of the core mechanisms underlying their ruminal modulatory effects. It has been hypothesized that saponins might indirectly reduce methanogenesis through the inhibition of protozoa, given that a substantial proportion of ruminal methanogens are associated with protozoal surfaces [63]. Studies have reported that saponins decreased methane production by 14.8% in vitro [64] and by 37% in vivo [65]. A meta-analysis indicated that, numerically, yucca saponins showed greater methane-mitigating efficacy than tea saponins, and were also superior to quillaja saponins [66]. However, contrasting findings have also been reported, as yucca saponins increased the propionate proportion in vitro but did not result in a reduction in methane production [67]. Overall, the methane-reducing effects of saponins are highly variable, with in vitro studies generally demonstrating higher mitigation amplitudes (ranging from 14.8% to 39%) [66], whereas in vivo responses are more moderate (6% to 27%) [68], which may be attributable to differences in saponin source, dosage, animal species, and ruminal microbial adaptation [69]. In addition, saponins have been shown to modulate rumen microbial populations, with inhibitory effects on certain cellulolytic bacteria and fungi, although responses are species- and dose-dependent [70]; moreover, they can divert metabolic hydrogen from methanogenesis toward propionate formation [71].

The efficiency of feed conversion into livestock and poultry products is generally low, with 50% to 80% of dietary nitrogen being excreted in manure [72]. Upon microbial decomposition of these excreta, ammonia is released, making feces and urine the primary sources of ammonia emissions [73,74]. This not only causes malodorous pollution, but also compromises animal health and production performance, whereas YSE may reduce methane production by indirectly inhibiting ruminal methanogens and modifying rumen fermentation patterns [75]. Wu et al. [38] demonstrated that supplementing liquid poultry manure with 0.5% YSE significantly reduced cumulative NH_3_ emissions by 20.18% during 60 days of storage. This mitigation was associated with the suppression of key ammonifying bacteria (e.g., *Proteiniphilum*) and the reduction in NH_4_^+^-N accumulation, alongside the ammonia-binding capacity of YSE glycocomponents.

**Figure 1:**
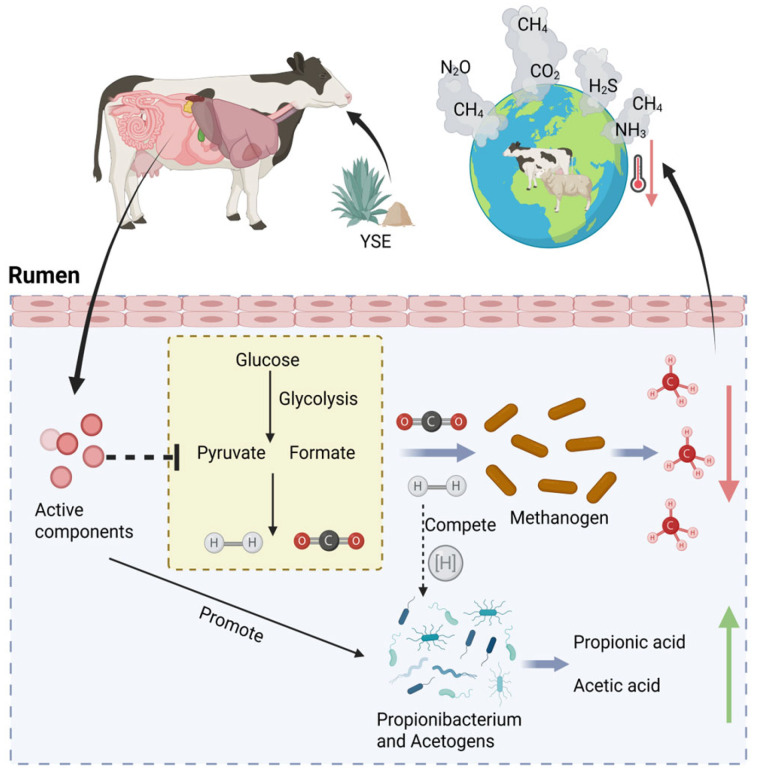
Schematic diagram of the mechanism by which YSE regulates ruminal fermentation and inhibits rumen methanogenesis in ruminants. Red arrows: decrease; green arrows: increase. Created in Biorender. Shiqi Xue. (2026) https://BioRender.com.

Similarly, Zúñiga-Serrano et al. [19] compiled that steroidal saponins in YSE can inhibit the growth of ammonia-producing bacteria (e.g., *Fusobacterium necrophorum* and *Clostridium perfringens*) and ruminal protozoa, likely by disrupting microbial cell membranes, which may reduce excessive protein degradation and ammonia production; additionally, the water-soluble non-butanol fraction of YSE has been implicated in nitrogen metabolism modulation, and the saponin components are known to trap ammonia nitrogen, contributing to lower ruminal ammonia concentrations. Further investigations have revealed that increasing dietary YSE linearly reduced the maximum rumen ammonia N concentration, and proposed that this effect is mediated by the direct ammonia-binding capacity of the glycol component of saponins, alongside a reduction in ciliate protozoa that limits microbial protein breakdown in the rumen [44]. The mechanism by which YSE regulates ruminal fermentation and methane emission is shown in Figure 1.

### 4.3. Maintenance of Gut Health

YSE promotes intestinal health via enhancing barrier function, modulating gut microbiota, and exerting anti-inflammatory and antioxidant effects [76]. Studies have demonstrated that YSE ameliorates intestinal barrier function in weaned piglets by upregulating the expression of tight junction proteins and mucins [77]. When co-supplemented with synbiotics, it reduces the concentrations of pro-inflammatory cytokines (IL-2, IL-6, TNF-α) in the serum of laying hens [78]. In mirror carp, dietary YSE upregulated Nrf2 and downregulated Keap1 mRNA expression, accompanied by increased T-AOC and decreased MDA levels, suggesting that YSE enhances intestinal antioxidant capacity through the Nrf2/Keap1 pathway [39,79]. Animal studies have demonstrated that YSE can selectively inhibit nitrifying bacteria, *Escherichia coli*, and ammonifying bacteria, thereby reducing the production of ammonia nitrogen and nitrite in the hindgut [80,81]. Meanwhile, it promotes the proliferation of beneficial bacteria such as *Lactobacillus*, *Streptococcus*, *Parabacteroides* and *Desulfobulbus*, thereby regulating the balance of gut microbiota [82,83]. Combined with *Clostridium butyricum*, it increased Ruminococcaceae, decreased Pseudomonadaceae and S24-7, improved villus height and villus-crypt ratio in weaned rabbits [84].

**Figure 2:**
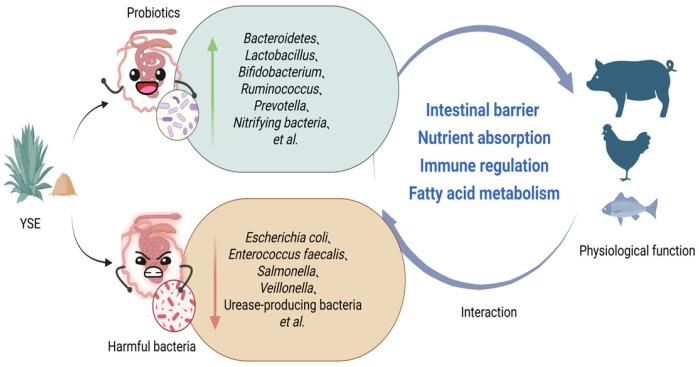
Regulatory mechanism of YSE on intestinal microbiota and host physiological performance. Red arrows: decrease; green arrows: increase. Created in Biorender. Shiqi Xue. (2026) https://BioRender.com.

Canine studies demonstrated that YSE reduced fecal ammonia and putrescine concentrations, elevated levels of short-chain fatty acids (SCFAs) such as propionate, and improved fermentation patterns [85]. In meat pigeons, 4-Hydroxyphenylacetic acid (4-HPAA, from YSE) at 500–2000 mg/kg elevated serum IgG and IgM levels and increased immune organ indices, indicating enhanced humoral immunity [11]. The same study also observed upregulated *CAMK2G* and *MKNK1* gene expression in the jejunum, with *CAMK2G* being associated with intestinal epithelial cell protection. However, a direct causal link between these gene expression changes and the immunomodulatory effects has not been established. The mechanism by which YSE modulates gut microbiota is shown in Figure 2.

## 5. Application in Livestock, Poultry and Aquatic Animals

### 5.1. Monogastric Animals

#### 5.1.1. Swine

The core biological functions of YSE are primarily manifested in two key domains: intestinal health maintenance and nitrogen metabolism regulation. Specifically, YSE significantly enhances systemic and local antioxidant capacity, as evidenced by increased activities of T-AOC, SOD, GSH-Px and CAT, alongside decreased levels of oxidative damage markers such as MDA in both serum and intestinal mucosa [39,77,86,87,88]. This effect is mechanistically mediated via the activation of the Nrf2/Keap1 signaling pathway, with YPB identified as the primary contributory bioactive component [39]. Furthermore, YSE improves intestinal morphological architecture, characterized by increased villus height and an elevated villus height-to-crypt depth (V/C) ratio, while simultaneously upregulating the expression of tight junction proteins (Occludin and ZO-1). Concurrently, it exerts a favorable modulation of the gut microecology, promoting the proliferation of beneficial genera such as *Lactobacillus*, *Bifidobacterium*, and *Prevotella*, while suppressing potentially pathogenic taxa including *Escherichia coli* and Proteobacteria [77,86,87,89]. Additionally, YSE administration effectively reduces serum urea nitrogen and intestinal ammonia nitrogen concentrations [77,86,88,90,91], and curtails the emission of noxious gases, including NH_3_ and H_2_S [88,91,92]. The underlying mechanisms for these nitrogen-related effects are attributed to the inhibition of urease activity, the binding of ammonia nitrogen, and the subsequent improvement in protein utilization efficiency [88,92].

However, the effects of YSE on growth performance (ADG, ADFI, F/G) have shown marked discrepancies across studies. Yang et al. [86] and Sampath et al. [91] reported that YSE improved ADG and ADFI, whereas Fan et al. [77], Santoru et al. [90], Gebhardt et al. [93], and da Silva et al. [89] all reported no significant changes. Moreover, da Silva et al. [89] even indicated that dietary supplementation with a high dose of YSE (250 mg/kg) in weaned piglets reduced nutrient digestibility and goblet cell count, while Gebhardt et al. [93] found that a low dose of YSE (62.5 mg/kg) in finishing pigs had negative effects on G: F and weight gain. Possible reasons include: (i) Animal physiological stage, as weaned piglets have a fragile intestinal barrier and high oxidative stress, so the antioxidant and barrier-repairing effects of YSE are more likely to be evident [77,86,89]; whereas finishing pigs have a mature digestive system, leaving limited room for improvement [90,93]. (ii) Active saponin content, given that a product at 300 mg/kg provides only 32 mg/kg saponins (approximately 10%), which may be below the effective threshold [90], while products with higher active ingredient contents of 30% and 60% showed superior effects [77,86]. (iii) Synergy with combined additives, since YSE combined with AZn and β-mannanase improved growth performance [91], and YSE combined with enzyme complex improved FCR [89], but YSE alone worsened FCR [93], suggesting that its biological benefits are synergy-dependent. (iv) Basal diet differences, because high-digestibility diets (containing whey powder and full-fat soybeans) may mask the improvement potential of YSE [89], particularly typical European finishing diets [90], while YSE effects were more pronounced in simple diets [77,86].

Furthermore, Fan et al. [77] confirmed that YSE upregulated the mRNA expression of *MUC-2*, whereas da Silva et al. [89] reported decreased *MUC-2* mRNA expression in YSE-supplemented groups, which may be attributable to the higher digestibility of the basal diet in the latter study (reducing mucosal stress-induced compensatory responses) or to differences in YSE dosage and product formulation. Dose–response studies on YSE in swine production remain relatively scarce, and further investigations with graded dose titrations are warranted. The recommended dosage for weaned piglets appears to be in the range of 120 mg/kg [77], while for gestating-lactating sows, 600 mg/kg was optimal for reproductive performance [88]. In practical production, YSE should be positioned as an intestinal health maintenance agent and a nitrogen emission reduction tool rather than a growth promoter [77,86,88,90,91,92]. Its application should be prioritized during stress windows, such as the weaning period and the periparturient phase in sows [77,86,87,88]. It is advisable to select products with clearly defined saponin content and standardized composition, and to adopt compound formulations (in combination with enzymes, zinc, or chromium) to amplify the beneficial effects [89,91,93]. Monitoring parameters should focus on fecal ammonia odor, serum urea nitrogen, and antioxidant indices, rather than over-relying on body weight gain data [39,77,86,87,88,90,91]. Ultimately, YSE should serve as an adjunctive tool for environmental and intestinal health management in pig operations, integrated with low-protein diets and precision feeding techniques, to jointly achieve the dual objectives of cost reduction, efficiency improvement, and emission mitigation. Effects of YSE supplementation in swine are shown in Table 2.

#### 5.1.2. Poultry

YSE has demonstrated clear and consistent positive effects in broiler and laying hen production, primarily manifested in the following five dimensions. First, it improves growth performance and feed efficiency. Multiple studies have consistently reported that Yucca supplementation reduces feed conversion ratio (FCR) by 5–9% and increases body weight gain by 4–8% [83,94,95,96,97], with positive responses observed across various breeds, including yellow-feathered broilers [95], Ross 308 [83,96], and Cobb 500 [98,99]. Second, it enhances humoral immunity and anti-inflammatory capacity. Dai et al. [95] and Mao et al. [100] confirmed that Yucca powder significantly elevated serum IgA, IgM, and IgY levels while reducing pro-inflammatory cytokines such as IL-6 and TNF-α. Furthermore, in a coccidia challenge model, a combination product of *Quillaja saponaria* and *Yucca schidigera* (MPU) was found to reduce the overexpression of IFN-γ and IL-10 in the jejunum, suggesting an immunomodulatory rather than a purely activating effect [101].

Third, it enhances the antioxidant defense system. Several studies have shown that Yucca-treated groups exhibited significantly increased SOD, GSH, and T-AOC, along with decreased MDA levels [95,98,99], and the mechanism may be related to the free radical scavenging activity of resveratrol and yuccaols present in the extract [95,99]. Fourth, it optimizes the gut microecology. Yucca or YSE reduced the counts of harmful bacteria such as *Escherichia coli* (by 20–60%) and increased the abundance of beneficial bacteria such as *Lactobacillus* [78,83,98,99], while also improving the villus height-to-crypt depth ratio in the small intestine [95,100]. Fifth, it reduces ammonia emissions and environmental load. Multiple studies have confirmed that YSE decreases litter nitrogen content by 20–46% and in-house ammonia concentrations by 16–36% [102,103,104,105,106], and this effect is likely directly associated with the ammonia-binding capacity and urease inhibitory activity of Yucca saponins [105].

However, divergent views exist regarding the comparative efficacy of YSE versus anticoccidial drugs, as well as its effects on feed intake, laying rate, and egg quality. Kozłowski et al. [96] reported that 500 mg/kg YS was superior to the narasin + nicarbazin anticoccidial combination in reducing oocyst shedding (OPG: 114 in the YS group vs. 243 in the anticoccidial group), whereas Curry et al. [107] indicated that the nicarbazin-based anticoccidial group outperformed the plant additive combination (containing oregano oil, citrus oil, and YSE) alone in terms of body weight gain and FCR. Possible reasons include substantial differences in saponin content—the former product contained ≥10.5% saponins, while the YSE proportion in the latter was unspecified and total saponins were likely lower—and the distinct modes of action: ionophore anticoccidials act through direct killing of Eimeria [108], whereas YSE primarily exerts its effects via saponin binding to cholesterol in the parasite cell membrane, leading to loss of membrane integrity [96,101], a process that is influenced by intestinal pH, saponin solubility, and feed matrix, resulting in greater variability in efficacy.

Mert [83] and Sahoo et al. [94] observed reduced FI in the YSE groups, while Dai et al. [95] and Munezero et al. [106] reported increased FI. The reduction in FI may be attributed to improved nutrient digestibility by YSE [83,94,106], such that animals do not need to consume more feed when diets are isocaloric and isonitrogenous. The increase in FI may be due to YSE enhancing intestinal digestive and absorptive function, and the multi-carbohydrase complex eliminating the anti-nutritional effects of non-starch polysaccharides [95,106]; additionally, low-protein diets (CP 15–17%) may induce compensatory feed intake due to protein deficiency, thereby promoting consumption [106]. Song et al. [78] supplemented laying hen diets with a synbiotic + YSE compound and found a significant increase in laying rate (from 94.6% to 97.0%), whereas other studies reported no difference in laying rate between YSE treated and control groups [97,100]. This suggests that the improvement in laying rate may be primarily attributable to the probiotics (*Lactobacillus plantarum* + *Bacillus subtilis*) rather than YSE alone. Furthermore, it is possible that neither an overly prolonged (20 weeks) [97] nor an overly short (6 weeks) [100] YSE supplementation period allows observable improvements in eggshell quality, whereas a moderate supplementation period (12 weeks) may be required to effectively enhance laying rate.

Therefore, in practical production, farmers should select the type and dosage of YSE products according to their production objectives. If the primary goal is growth promotion and FCR reduction (in broilers aged 1–42 days), YSE powder (rather than pure saponin extract) is recommended at a dosage of 100–125 mg/kg [83,94,95,96,109], as this regimen has shown optimal effects on intestinal morphology and immune parameters. If the target is anticoccidial, anti-stress, and intestinal protection, a standardized product with high saponin content (≥10.5%) is recommended at a dosage of 500 mg/kg [96,100,101,103], and its efficacy is greatest under mild-to-moderate challenge conditions, whereas anticoccidial drugs are still required under severe challenge [107]. For ammonia emission reduction, supplementation via drinking water at 0.5–1 mL/L (8 h/day) or via feed at 100–125 mg/kg is effective [99,102]. Furthermore, YSE can serve as a core component of antibiotic-alternative strategies; when combined with humic acid, probiotics, or *Quillaja saponaria* bark, multiple parameters (intestinal immunity, antioxidant status, and FCR) are significantly improved compared with YSE alone. It is recommended to incorporate YSE into an integrated antibiotic-replacement regimen (YSE + acidifiers + probiotics), rather than relying on YSE as a standalone product to address all intestinal health issues. Effects of YSE supplementation in poultry are shown in Table 3.

### 5.2. Ruminants

Multiple studies have consistently reported that YSE supplementation exerts no significant effect on dry matter intake (DMI) [111,112,113,114,115], indicating that YSE does not influence production performance through appetite regulation, and that its effects may stem from alterations in nutrient utilization efficiency rather than changes in feed intake. Regarding ruminal fermentation patterns, YSE supplementation shifts the fermentation type toward a propionate-type pattern, with multiple studies [112,116,117] observing an increased propionate proportion and a decreased acetate-to-propionate ratio, which is consistent with the mechanism whereby saponins inhibit protozoa, reduce hydrogen sinks, and promote the proliferation of propionate-producing bacteria. Wolschick et al. [116] further confirmed, via 16S sequencing, the enrichment of fiber-/starch-degrading bacteria such as *Xylanibacter* and *Lentimicrobium*, while Johnson et al. [118] reported an increased abundance of *Prevotella ruminicola*. Studies have shown that dietary YS supplementation increased IgA/IgG levels and reduced diarrhea incidence in calves [114], and that YSE supplementation decreased the need for antibiotic treatments in stressed cattle [111], collectively indicating the immunomodulatory potential of Yucca, with this effect being more readily apparent under high-stress conditions. Ruminal pH and total VFA concentrations remained unaffected, which is also a common finding across most in vivo studies [112,116,118], suggesting that YSE does not cause disruptive interference with the basal ruminal environment.

However, divergent views exist regarding the effects of YSE on production performance. Dietary YSE supplementation linearly improved feed efficiency in Angus × Hereford calves [111], Angus crossbred heifers [112], and Awassi lambs (low-dose group) [115], whereas decreased feed efficiency was observed in Holstein calves and Awassi lambs (high-dose group) [115,119]. These discrepancies may be related to the inflammatory-immune balance [111,119], ruminal fermentation patterns and propionate production efficiency [112,119], intestinal barrier integrity [111,112,119], and YSE dosage [115]. This also suggests that caution should be exercised when using YSE in young animals. In terms of nitrogen metabolism, the review by Patra et al. [117] and the study by Alsubait et al. [115] consistently reported that YSE supplementation reduced ammonia nitrogen, whereas Yi et al. [113] observed simultaneous increases in NH_3_-N and TVFA in the YSE group in a long-term trial with Angus steers. This may be attributable to accelerated microbial protein synthesis accompanied by greater accumulation of nitrogen degradation intermediates under high fermentation rates [113], rather than a simple ammonia-binding effect of saponins [117]. Furthermore, YSE reduced MDA/ROS and exhibited anti-inflammatory effects in calves [114] and Jersey cows [116], but significantly increased ROS/TBARS and inflammatory cytokines (TNF, IL-1, IL-6) in growing calves [119]. It is speculated that this may stem from intestinal malabsorption caused by local inflammation, which serves as a trigger for reduced feed efficiency [119]. In brief, YSE acts both as an antioxidant [114] and as a potential pro-inflammatory stimulant (due to the membrane-lytic activity of saponins) [117], and its actual effects depend on dosage and animal developmental stage.

Furthermore, in lactating dairy cows, daily supplementation with 5, 15, or 30 g of YSE linearly reduced the peak ruminal ammonia nitrogen concentration (from 228 mg/L to 179 mg/L), increased the propionate proportion, and decreased the butyrate proportion, while acetate remained unchanged. Methanogen abundance decreased with increasing supplementation levels, and milk fat content increased from 38.9 g/kg to 42.0 g/kg [44]. Acetate promotes lipogenesis and is closely associated with milk fat synthesis [120]. Although acetate did not change significantly in this trial, the increase in milk fat may be attributed to enhanced insulin signaling and increased mammary glucose supply mediated by elevated propionate [121]. In addition, the decreasing trend in butyrate may reflect its rapid conversion to β-hydroxybutyrate, which can serve as an alternative primer carbon source for de novo milk fat synthesis [122]. The combined effects of these two pathways synergistically support milk fat synthesis.

Nevertheless, another study reported that daily supplementation with only 5 g of YSE reduced milk protein from 34.9 g/kg to 32.8 g/kg, and when combined with yeast, it decreased dry matter intake. Both supplementation modes downregulated methanogens and increased the abundance of *Prevotella*, but ruminal ammonia nitrogen and nitrogen excretion remained unchanged [118]. This may be due to excessive binding of free ammonia by YSE components, limiting the nitrogen source available for microbial protein synthesis [117], or the slight bitterness of saponins may impair palatability [123], ultimately resulting in impaired milk protein deposition. In addition, a sheep reproduction trial showed that YSE inhibited follicular development and reduced progesterone and estradiol; this negative reproductive effect has not been reported in cattle, suggesting species specificity [124].

In practical production, YSE represents a reliable approach for environmental emission reduction. In lambs, YSE at 600 mg/kg DM significantly reduced ammonia and H_2_S emissions from feces and urine [115]. Nano-encapsulation technology has demonstrated strong methane inhibition potential in vitro, but its cost-effectiveness and in vivo conversion efficiency remain to be validated [125]. YSE-containing compound additives may possess synergistic potential; however, the independent contribution of YSE needs to be evaluated by decomposing the components of each group. Current evidence does not support its promotion as a sole additive across various cattle herds. Moreover, YSE should not be indiscriminately used as a substitute for antibiotic growth promoters. In conventional finishing cattle and lactating cows, YSE supplementation resulted in no improvement in production performance or even a decrease in milk protein, indicating that it cannot replicate the consistent weight gain effects of ionophores such as monensin [118,126,127]. It is recommended that producers clearly define their objectives (disease resistance, emission reduction, or growth promotion) prior to use and select the corresponding dosage and product form accordingly. Effects of YSE supplementation in ruminants are shown in Table 4.

### 5.3. Aquatic Animals

The application of YSE in aquaculture has demonstrated highly consistent beneficial effects, including reductions in ammonia nitrogen, enhanced antioxidant defense, improved immune function, and better growth performance. Multiple studies have consistently reported that YSE significantly reduces total ammonia nitrogen and ionized ammonia concentrations [80,128,129,130,131,132,133,134,135,136,137,138,139], and this effect is attributed to the ammonia-binding capacity of steroidal saponins and the surface activity of polysaccharide components in YSE [128]. At the antioxidant level, YSE increased SOD activity and decreased MDA levels [128,129,130,131,134,135,136,137], suggesting that YSE may enhance endogenous antioxidant defense through free radical scavenging and activation of the Nrf2-ARE pathway [137]. In terms of immune parameters, lysozyme activity, complement activity, and phagocytic activity were consistently improved across multiple studies, indicating that YSE can systematically strengthen the non-specific immune barrier in fish [128,129,130,131,132,133,134,135,136]. Furthermore, YSE reduced the feed conversion ratio [80,128,129,130,131,132,133,134,135,136,138,139,140], which was closely associated with enhanced digestive enzyme activities (protease, amylase, and lipase) [128,129].

Although the core benefits of YSE are highly consistent, the performance of specific indicators has shown marked divergence, primarily in three aspects. First, the bidirectional regulation of pro-inflammatory cytokine expression. Under normal physiological conditions, Elsisy et al. [128], Barducci et al. [129], and Njagi et al. [134] all reported that YSE or Bacillus upregulated the expression of TNF-α, IL-1β, and IL-8, reflecting an immune-enhancing effect; however, under stress conditions (ammonia exposure and heat stress) [130,137,140], these factors were significantly downregulated, reflecting an anti-inflammatory protective effect. This bidirectional modulation precisely demonstrates that the immunohomeostatic function of YSE maintains balance rather than unidirectional stimulation. Second, discrepancies in growth-promoting effects. Both Bae et al. [133] and Njagi et al. [134] used the same dosage (0.1%) of the commercial YSE product (De-Odorase^®^), but the former found no growth-promoting effect in Japanese eel, while the latter observed effective growth promotion in Nile tilapia, which may be related to differences in animal species and supplementation duration. Third, the dominant effect is determined by stress type. In ammonia stress models [130,131,140], the core effect of YSE was to reduce ammonia concentrations in water, thereby alleviating toxicity; in heat stress models [137], the core effect was to activate the Nrf2/Hsp70 pathway to alleviate endoplasmic reticulum stress; in chemically induced models [141], the phenolic compounds in YSE primarily exerted neuroprotective and antioxidant effects. This suggests that the targets and mechanisms of YSE vary depending on the type of stress.

In summary, the recommended dietary supplementation dosage ranges from 0.1% to 0.2% (or 1–2 g/kg), and the water supplementation dosage ranges from 0.75 to 1 mL/L or 8–10 mg/L, depending on water exchange frequency. Secondly, YSE should be preferentially applied in high-density culture, high-temperature seasons, or production systems prone to excessive ammonia nitrogen, as its cost-effectiveness in ammonia-binding and anti-stress effects is superior to that of probiotics or zeolite alone [135]. Furthermore, the combination of YSE and Bacillus exhibits synergistic effects [80,128,130,139], possibly because YSE reduces environmental ammonia nitrogen pressure, creating favorable conditions for probiotic colonization, while digestive enzymes and antimicrobial peptides secreted by Bacillus compensate for the deficiency of YSE in direct bactericidal activity. Finally, the sensitivity to YSE varies among different aquatic species. Sea bass and tilapia show optimal responses at 0.1–0.2% supplementation levels, whereas Japanese eel [133] and zebrafish [141] exhibit relatively milder responses, suggesting that dosage should be optimized in a species-specific manner, and graded dose trials are recommended prior to practical application. Notably, studies on YSE in shrimp and other commercially important aquatic species (e.g., crabs, crayfish) remain scarce over the past decade, and most existing evidence is derived from controlled laboratory trials rather than commercial-scale production systems, underscoring the urgent need for more validation studies under practical farming conditions. Future research should focus on the differential mechanisms of YSE active components (saponins vs. phenolic compounds) under various stress models, as well as the effects of long-term feeding on intestinal microbiota and flesh quality. Effects of YSE supplementation in aquatic animals are shown in Table 5.

### 5.4. Comparison of YSE’s Cross-Species Application Effectiveness

Extensive research has been conducted on the application of YSE in various livestock, poultry, and aquatic animals; however, significant differences exist in research depth and effect characteristics among these species over the past decade. The optimal YSE supplementation levels, primary effects, proposed mechanisms, and research limitations for different species are summarized in Table 6. Although these summary tables can serve as a reference for the range of YSE inclusion levels, the dose–response data available from existing studies remain very limited for most animal species. The current evidence is still insufficient to define a uniform supplementation level that is applicable across all species, or even within a single species. For young animals, the appropriate inclusion level is particularly uncertain, and further dose–response trials are urgently needed to clarify this issue.

Cross-species comparisons reveal that the mechanisms of action of YSE share certain common features, but also exhibit species-specific pathways arising from differences in physiological structure. In terms of common mechanisms, YSE primarily inhibits urease activity and binds ammonia molecules through its saponin components, thereby reducing ammonia emissions from feces and urine—an effect that has been validated in pigs [77,88], poultry [102], ruminants [123], and fish [128,130]. Meanwhile, YSE enhances the intestinal physical barrier by upregulating the expression of tight junction proteins (ZO-1 and Occludin) and activates the Nrf2 antioxidant pathway to alleviate oxidative stress, mechanisms that have been demonstrated in weaned piglets [39,77], broilers [95], and rainbow trout [129]. However, species-specific mechanisms are equally prominent: in ruminants, the primary target of YSE is rumen protozoa, and its partial defaunation effect alters the ruminal fermentation pattern (by increasing the propionate proportion and reducing methane production) [117]; in monogastric animals, YSE primarily acts by modulating the hindgut microbiota composition (by increasing *Lactobacillus* and *Bifidobacterium*, while reducing *Escherichia coli*.) [86,87]; in aquatic animals, YSE also significantly improves water ammonia nitrogen and nitrite concentrations, a unique application scenario not observed in terrestrial animals [80,138]. Furthermore, the inhibition of follicular development and downregulation of sex hormones observed in luteal-phase ewes [124] is opposite to the effect of promoting estrus and ovarian function recovery in sows [88], suggesting that the hormonal regulatory effects of saponins may be species-selective.

Despite the promising cross-species application prospects of YSE, comparative and safety data from current studies remain critically insufficient. From a dose–response perspective, the optimal supplementation levels vary considerably among species, and systematic dose-gradient trials to determine toxicity thresholds are lacking. Existing evidence indicates that supplementation exceeding 250 mg/kg in weaned piglets may exert negative effects [89], that 600 mg/kg DM in lambs suppresses growth performance [115], and that 188 mg/kg DM in calves, while altering ruminal fermentation, induces systemic inflammatory responses [119], suggesting marked differences in tolerance to excessive YSE among species. Long-term safety evaluations are currently limited to short-term trials of at most a few months in duration, and no cross-generational or full-production-cycle safety data are yet available.

**Figure 3:**
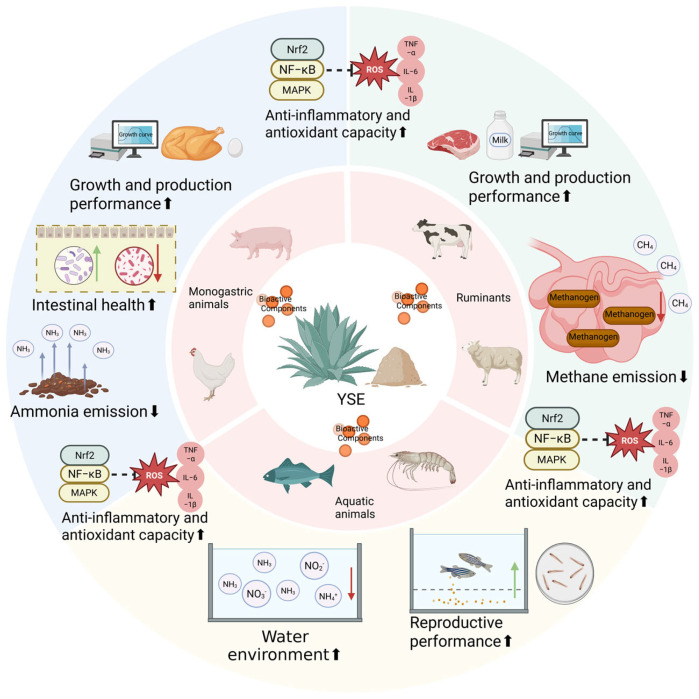
Application of YSE in livestock, poultry and aquatic animals. Red arrows: decrease; green arrows: increase. Created in Biorender. Shiqi Xue. (2026) https://BioRender.com.

From a regulatory perspective, YSE is recognized as Generally Recognized as Safe (GRAS) by the U.S. Food and Drug Administration (FDA) under Title 21 of the Code of Federal Regulations, Section 172.510, and may be added to animal feeds as a flavoring agent at a maximum inclusion level of 125 mg/kg in complete feed [143,144]. In the European Union, YSE is currently approved as a feed additive under the functional category of sensory compounds (flavoring agents) for all animal species, but is subject to re-evaluation in accordance with Regulation (EC) No. 1831/2003. Additionally, the European Food Safety Authority (EFSA) has established a safe dosage of 250 mg/kg complete feed for Magni-PHI^®^ (containing 15% Yucca), and recommends a maximum saponin content of 5.1% in the additive specification [23,145]. The Ministry of Agriculture and Rural Affairs of China has included YSE in the Catalogue of Feed Additives, permitting its use in all categories of animal production [146]. However, there is currently no globally harmonized maximum limit standard for saponin content in YSE. The applications of YSE in livestock, poultry and aquatic animals are summarized in Figure 3.

## 6. Future Perspectives

Although research on YSE has made considerable progress, several critical knowledge gaps must be addressed to translate experimental findings into evidence-based commercial applications.

First, standardization of commercial YSE products. Crude extracts currently vary markedly with plant origin, harvest season, processing techniques, and product formulation, leading to poor comparability of results across studies. High-resolution mass spectrometry and nuclear magnetic resonance (NMR) should be employed to establish robust qualitative and quantitative methods for marker compounds (e.g., individual saponins, polyphenols, and polysaccharides), thereby promoting product standardization and enabling meaningful cross-study comparisons.

Second, dose optimization and species-specific protocols. The effects of YSE are clearly dose-dependent and stage-specific; for example, high doses may suppress growth in calves while promoting weight gain in finishing cattle, and the optimal supplementation level for weaned piglets (approximately 120 mg/kg) differs substantially from that for gestating sows (600 mg/kg). Systematic dose–response studies across species, physiological stages, and dietary backgrounds are urgently needed to establish precise application protocols and identify toxicity thresholds. Such studies should incorporate both performance and safety endpoints, including histopathological examination and serum biochemical profiling.

Third, long-term safety and multi-generational effects. Current safety evaluations are largely limited to short-term trials of at most a few months in duration. Comprehensive long-term feeding studies covering full production cycles, as well as multi-generational assessments, are required to evaluate potential cumulative effects, tissue residues, and impacts on offspring health and development. These data are essential for regulatory approval and consumer acceptance.

Fourth, mechanistic elucidation at the molecular level. Although mechanistic studies have focused on anti-inflammatory and antioxidant pathways (Nrf2/ARE and NF-κB), the specific molecular targets of individual YSE bioactive fractions, their receptor interactions, and the cross-species differences in metabolic regulation remain poorly understood. Integrated omics approaches (transcriptomics, proteomics, and metabolomics) combined with gene-knockout or receptor-inhibition models are recommended to unravel the complex molecular networks underlying YSE’s pleiotropic effects.

Fifth, interactions with other feed additives. Given that many positive outcomes have been observed when YSE is combined with probiotics, enzymes, organic acids, or zinc, systematic studies on additive and synergistic interactions are warranted. Such research should employ factorial designs to delineate the independent contribution of YSE versus interactive effects, enabling the rational design of multi-component feed additive formulations.

Sixth, commercial-scale validation and economic feasibility. While the efficacy of YSE in reducing ammonia, hydrogen sulfide, and methane emissions is well documented in laboratory and experimental farm trials, evidence from large-scale commercial operations remains critically insufficient. Multi-site, long-term field trials are needed to evaluate the consistency and magnitude of these environmental benefits under real-world production conditions. Concurrently, comprehensive economic analyses, including cost benefit assessments, feed cost savings, mortality reduction, and potential revenue from carbon credits, should be conducted to determine the net economic value of YSE supplementation across different production systems.

Seventh, life-cycle assessment and environmental sustainability. Beyond direct gas emission measurements, life-cycle assessment (LCA) should be incorporated to systematically evaluate the full environmental footprint of YSE application, including its impacts on carbon and nitrogen footprints, water use, land use, and overall ecosystem health. Such assessments would provide robust data to support policy formulation for low-carbon livestock production and identify potential environmental trade-offs that may not be apparent from single-indicator studies.

Eighth, precision livestock farming integration. The integration of YSE application with precision livestock farming technologies, such as real time monitoring of ammonia concentrations, feed intake, and health status, offers opportunities for dynamic and individualized supplementation strategies that optimize efficacy while minimizing waste and cost. Research on sensor-based decision support systems for YSE administration is warranted.

In summary, YSE, as a multifunctional, low-residue green feed additive, has already established a solid research foundation and holds promising application prospects. However, realizing its full potential requires interdisciplinary research efforts aimed at standardizing its bioactive components, elucidating its mechanisms of action, refining application protocols, validating performance under commercial conditions, and assessing its economic and environmental sustainability. These endeavors will provide a robust theoretical and practical basis for the scientific promotion of YSE in ecological livestock production.

## 7. Conclusions

As a widely available plant-derived feed additive with abundant bioactive components, *Yucca schidigera* extract (YSE) exhibits considerable application potential in the development of green low-carbon livestock production. This paper systematically reviews the primary bioactive components of YSE (steroidal saponins, polyphenols, and polysaccharides), its core mechanisms of action (anti-inflammatory activity, antioxidant activity, regulation of ruminal metabolism and ammonia nitrogen metabolism, and maintenance of intestinal health), and its specific application effects in pigs, poultry, ruminants and aquatic animals. Research suggests that YSE exerts biological functions via multiple targets. On the one hand, steroidal saponins and polysaccharides can bind ammonia nitrogen and inhibit urease activity, thereby reducing the emission of malodorous gases from livestock production at the source; this effect has been verified across multiple animal species under both laboratory-controlled and experimental farm conditions. On the other hand, YSE alleviates oxidative stress and inflammatory responses by activating the Nrf2 signaling pathway and inhibiting the NF-κB pathway, while improving intestinal barrier integrity and regulating intestinal microbiota composition, which ultimately enhances animal growth performance, reproductive efficiency, and product quality. Nevertheless, it should be noted that most currently reported beneficial effects of YSE, especially those related to methane emission mitigation and production performance improvement, are obtained from trials under controlled experimental conditions. The magnitude and stability of these effects under large-scale commercial production conditions still require systematic validation, and the level of research evidence varies significantly across animal species, YSE dosages, and experimental designs.

## Figures and Tables

**Table 1:** Main bioactive components, biological functions and corresponding action sites of YSE.

Raw Material Source	Main Bioactive Compounds	Biological Functions	Action Sites	Refs
Yucca stem meal	Saponins (10%)	Antiprotozoal activity; Serum cholesterol reduction; Regulation of intestinal mucosal permeability; Suppression of Gram-positive bacteria	Intestinal epithelial cells; Peripheral blood; Ruminal microbiota	[5]
Yucca bark; Whole-plant Yucca powder	Bark: resveratrol (21.7 mg/g), yucca phenols A–E (72.6 mg/g) Whole plant: resveratrol (3.2 mg/g), yucca phenols A–E (10.0 mg/g)	Anti-inflammatory and antioxidant effects; Inhibition of platelet aggregation; Alleviation of arthritic inflammatory lesions	Macrophages; Synoviocytes; Chondrocytes; Platelets; Articular tissues	[5]
*Y. schidigera* juice A, B, C	Saponins (15%, 10%, 12%, respectively)	—	—	[8]
*Yucca* *schidigera* juice extract	Saponins (28.1% m/m via UV spectrophotometry; 29.9% m/m via HPLC-ELSD & gravimetric assay)	—	—	[10]
Yucca powder	Saponins (10%)	Anti-inflammation and antioxidation	—	[15]
YSE	Saponins (10.8%)	Reducing caecal ammonia nitrogen in vitro; Modulating caecal microbial fermentation; Regulating intestinal microbiota and mitigating ammonia emission in vivo	Intestinal microbial cell membranes; Gastrointestinal microbiota	[21]
YSE	Saponins (≥8%)	Antioxidation; Improving nutrient digestibility and absorption; Antiprotozoal activity; Ammonia abatement	Rumen microbes; Liver tissue; Intestinal tract; Serum metabolic system	[22]
Yucca powder	Saponins (12.5% by UV assay; 3% by HPLC)	Heat stress alleviation; Antioxidant; Thermoregulation improvement; Recovery of suppressed feed intake	Hypothalamic thermoregulatory and feed intake center; Plasma circulation; Duodenal mucosa	[30]
Yucca butanol extract	Saponins (30%)	Antibacterial and antiparasitic properties; Intestinal microbiota modulation	Intestinal pathogenic bacteria; Intestinal protozoa; Ruminal protozoa of ruminants; Intestinal mucosa	[20]
Yucca bark	Ultrasonic extraction: resveratrol (9.11 mg/g), 3,3′,5,5′-tetrahydroxy-4-methoxystilbene (9.10 mg/g) Soxhlet extraction: 3,3′,5,5′-tetrahydroxy-4-methoxystilbene (0.017 mg/g)	Anti-inflammatory and antioxidant effects; Inhibition of protein carbonylation and nitration; Attenuation of platelet lipid peroxidation	Vascular endothelial cells; Platelets; Pulmonary tissue; Articular tissue; Liver; Kidney	[19]
YSE	Saponins (30%, determined by UV spectrophotometry)	Ammonia emission reduction; Regulation of microbial community structure	Microorganisms	[38]
YSE (24 mg/L)	Yucca polysaccharide B (YPB, 1.872 mg/L), saponins (1.704 mg/L), resveratrol (26.4 μg/L)	Antioxidation; Alleviation of intestinal mucosal injury	Jejunal mucosa; Porcine small intestinal epithelial cells (IPEC-J2); Rat intestinal tissue	[39]
Yucca ethanol extract	Partial monomeric saponins, flavonoid glycosides	Moderate antioxidant activity; No in vitro antimicrobial activity against tested strains	—	[42]
YSE	Saponins (26.9 ± 0.15 mg/g), kaempferol (0.35 mg/g)	Anti-inflammatory and antioxidant activity	Gastric mucosa	[43]
YSE	Saponins (4.4%)	Antiprotozoal activity; Lowering caecal ammonia nitrogen; Modulating ruminal volatile fatty acid profiles; Mitigating methane production	Rumen protozoa; Ruminal bacteria; Methanogenic archaea	[44]
Yucca butanol extract	Saponins (25.43%)	Antioxidant and antibacterial capacity	—	[45]
Butanol fraction of Yucca extract (200 μg)	Saponins (30%), polyphenols (2.95%), flavonoids (7%)	Antioxidation and bacteriostasis	—	[46]
Aqueous fraction of Yucca extract (200 μg)	Saponins (1.81%), polyphenols (0.56%), flavonoids (0.165%)	Antioxidation and bacteriostasis	—	[46]

**Table 2:** Application effects of YSE supplementation in swine.

Animal Category	Experimental Design	Supplemental Dosage	Duration	Main Experimental Outcomes	Study Limitations	Refs
Weaned barrows (DLY crossbred, initial BW: 6.75 ± 0.30 kg)	Two groups with 14 pigs: CON (basal diet) YSE (+120 mg/kg YSE)	120 mg/kg diet (30% total bioactive components)	14 d	Improved intestinal morphology and barrier integrity; Enhanced nutrient digestibility and absorption; Elevated feed conversion ratio (FCR); Reduced ammonia emission	Single dosage level tested; Short supplementation period; The dominant bioactive fraction responsible for observed effects remains unclarified	[77]
Weaned piglets (Rongchang × Landrace × Large White crossbred, initial BW: 7.51 ± 0.54 kg)	2 × 2 factorial design, 40 piglets allocated into four treatments: CON (basal diet + 1 mL 0.85% saline) CU (+1 mL 1 × 10^9^ cfu/mL C. utilis in 0.85% saline) YSE (+1 mL 0.85% saline+120 mg/kg YSE) YSE + CU	120 mg/kg diet (60% total bioactive components)	28 d	Optimized intestinal morphology and growth performance; Alleviated diarrhea incidence; Enhanced systemic antioxidant capacity; Remodeled intestinal microbiota; Synergistic effects observed with combined additives	No antibiotic positive control group; Interactive effects of graded additive dosages not evaluated	[86]
Weaned piglets (initial BW: 6.43 ± 0.25 kg)	Six groups with 60 piglets: CD (control diet) CDY (+125 mg/kg YSE) CDE (+200 mg/kg enzyme complex) CDME (+400 mg/kg multienzyme complex with emulsifier) CDE + Y1 (+125 mg/kg YSE) CDE + Y2 (+250 mg/kg YSE)	125 mg/kg and 250 mg/kg diet (De-Odorase^®^; equivalent saponin contents: 65 mg/kg and 130 mg/kg diet, respectively)	32 d	Low-dose YSE exerts synergistic growth-promoting effects when combined with compound enzymes; Adverse physiological impacts detected under high YSE supplementation	Insufficient YSE dosage gradient; No antibiotic control; Independent biological effects of single additives cannot be distinguished in composite treatments	[89]
Weaned barrows (DLY crossbred, initial BW: 6.75 ± 0.30 kg)	In vivo animal trial: 14 barrows divided into CON and YSE groups (+120 mg/kg YSE) In vitro cell & rat oxidative stress model: purified yucca polysaccharide B (YPB) applied to IPEC-J2 porcine intestinal epithelial cells and rat models	In vivo: 120 mg/kg diet (30% total bioactive components) In vitro: 24 mg/L working solution containing 1.872 mg/L YPB	In vivo trial: 14 d	YPB activates PI3K/AKT-mediated NRF2 signaling axis to strengthen intestinal antioxidant defense and mitigate intestinal oxidative damage	Different administration routes between in vitro and in vivo assays; Linkage between YPB antioxidant function and nitrogen emission reduction not elaborated	[39]
Pigs from weaning to finishing stage (Landrace × Yorkshire × Duroc crossbred, initial BW: 6.57 ± 1 kg)	Three groups with 180 pigs: CON (Basal diet) TRT1 (+1000 mg/kg AZn + 700 mg/kg YSE + 500 mg/kg β-mannanase) TRT2 (+2000 mg/kg AZn + 700 mg/kg YSE + 500 mg/kg β-mannanase)	YSE fixed at 700 mg/kg within compound additive formulation	22 weeks (whole production cycle from weaning to slaughter)	Composite additive promotes growth performance, reduces harmful gas emissions, and elevates vaccine antibody titres; YSE can function as an immunostimulant feed additive	No single YSE treatment group; Cellular immunity and in vivo protective efficacy not measured	[91]
Finishing pigs Trial 1: PIC 337 × 1050 (initial BW: 27.3 ± 0.48 kg) Trial 2: PIC 359 × 1050 (initial BW: 29.3 ± 0.43 kg)	2 × 2 factorial arrangement, four groups with 1188 pigs (Cr: 0/200 μg/kg × YSE: 0/62.5 mg/kg), 1188 pigs 2 × 3 factorial arrangement, six groups with 2430 pigs (Cr: 0/200 μg/kg × YSE: 0/62.5/125 mg/kg)	62.5 mg/kg, 125 mg/kg diet (Micro Aid^®^ (Porterville, CA, USA) yucca-based feed concentrate)	117 d/103–113 d	Combined chromium and YSE slightly improves growth performance; Sole YSE supplementation generates no carcass quality benefits, and low-dose YSE exhibits adverse production effects; No synergistic interaction detected	Mechanisms underlying the negative impacts of low-dose YSE remain uncharacterized	[93]
Finishing pigs (Pietrain × Landrace × Large White crossbred, initial BW: 76.1 ± 5.2 kg)	2 × 2 factorial design, two groups with 40 pigs: CON (Basal diet) YSE (+300 mg/kg YSE)	300 mg/kg diet (Yucca-50^®^ (Anagalide S.A., Barbastro, Huesca, Spain) equivalent saponin content: 32 mg/kg diet)	42 d	300 mg/kg YSE supplementation does not alter growth performance or meat quality; Regulates whole-body nitrogen metabolism and improves nitrogen utilization efficiency; Safe for both barrows and gilts	Only single high dosage tested; Mechanisms of sex-specific response unclear; Limited saponin content restricts functional efficacy	[90]
Gestating and lactating Large White sows (parity: 4–6)	Four groups with 80 sows: CON (Basal diet) YSE600 (+600 mg/kg YSE) YSE1200 (+1200 mg/kg YSE) YSE2400 (+2400 mg/kg YSE)	600, 1200, 2400 mg/kg diet (≥10.5% saponins, ≥318 mg/kg resveratrol)	From d 80 of gestation to d 21 of lactation (~42 d)	Optimal dosage: 600 mg/kg YSE improves sow digestive function and reproductive performance; Higher dosages reduce fecal nitrogen and ammonia volatilization	Distinct functional mechanisms across graded dosages; Long-term effects on piglet development not investigated	[88]
Replacement gilts (Large White × Yorkshire crossbred, initial BW: 105 ± 15 kg)	Two groups with 20 gilts: CONT (Basal diet) YETG (+250 mg/kg YSE)	250 mg/kg diet (≥12% saponins)	35 d	Boosted nutrient digestibility and antioxidant capacity; Remodeled fecal microbiota to maintain systemic health status	Oestrus-related hormones and subsequent reproductive performance not monitored; Fecal microbiota cannot fully represent intestinal microbial profile	[87]

**Table 3:** Application effects of YSE supplementation in poultry.

Animal Category	Experimental Design	Supplemental Dosage	Duration	Main Experimental Outcomes	Study Limitations	Refs
Broiler chicks (Ross 308, initial BW: 38.38 ± 0.44 g)	Four groups with 240 broilers: C (basal diet) H (+300 mg/kg humate) Y (+100 mg/kg YS) HY (H + Y)	100 mg/kg diet	42 d	Combined humate and YS exerts synergistic effects; Optimizes growth performance, intestinal health and immune function of broilers; Shows potential as an antibiotic alternative	Independent effects of YSE cannot be isolated from composite formulation; Only one dosage level tested; Serum antioxidant and immune indices not determined	[83]
Broiler chicks	Two groups with 120 broilers: CON (basal diet) YS (+125 mg/kg YSE)	125 mg/kg diet	42 d	Improves growth performance, carcass traits and survival rate; Optimizes feed conversion ratio and reduces aggressive pecking behavior; Delivers favorable economic benefits	Single dosage tested; Serum biochemical parameters not measured; Active composition of YSE not characterized	[94]
Broiler chicks (yellow-feather broilers, 32.00 ± 2.00 g)	Four groups with 480 broilers: CON (basal diet) YSa (+500 mg/kg yucca saponin) YS (+500 mg/kg yucca power) QS (+500 mg/kg quillaiae cortex)	500 mg/kg yucca powder; 500 mg/kg yucca saponin (saponin ≥ 30%)	56 d	All three additives improve multiple production indices in broilers; Yucca powder achieves the best performance in feed conversion ratio, immunity and intestinal morphology	Only one dosage level tested for each product; Variation in saponin content across products limits direct comparison	[95]
Broiler chicks (Ross 308)	Four groups with 504 broilers: Non-challenged control Challenged control Challenged + 625 mg/kg Maxiban (anticoccidial drug) Challenged + 500 mg/kg Yucca Plus™	500 mg/kg Yucca Plus™ (saponin ≥ 10.5%)	35 d	YS alleviates coccidial-induced intestinal damage; Exhibits superior oocyst-suppressing efficacy to anticoccidial drugs; Can serve as a natural alternative	Single dosage tested; Dosage not equivalent between drug and yucca treatment groups	[96]
Broiler chicks (Ross 308, initial BW: 45.2 ± 0.3 g)	Four groups with 480 broilers: T1 (CP 21%) T2 (CP 19% + 0.02% YSE) T3 (CP 17% + 0.02% YSE) T4 (CP 17% + 0.02% YSE + 0.1% α-1, 6-galactosidase)	0.02% YSE (equivalent to 200 mg/kg diet)	35 d	Low-protein diets supplemented with YSE and compound enzyme do not promote growth; Enhance nutrient digestibility, carcass performance and reduce ammonia emission	Serum biochemical parameters not measured; Interactive effects between YSE and enzyme remain insufficiently studied	[106]
Broiler chicks (Ross 708)	Four groups with 128 broilers: Uninfected unchallenged control (UUC) Infected unchallenged control (IUC) IUC + 66 mg/kg salinomycin IUC + 110 mg/kg MagniPhi Ultra (Quillaja saponaria and *Yucca schidigera*)	110 mg/kg MagniPhi Ultra (8% total saponins)	Fed for 10 d prior to coccidial challenge; trial terminated at ~28 days of age post-challenge	MagniPhi Ultra improves intestinal barrier integrity and body weight gain; Reduces oocyst shedding and intestinal lesion scores; Modulates cytokine profiles with long-lasting efficacy	Single dosage tested; Independent contribution of yucca to the composite product is unknown	[101]
Broiler chicks	Five groups with 105 broilers: A (Positive control, high ammonia exposure) B (Negative control) C (+30 g/m^2^ potassium aluminum sulphate) D (+15 g/m^2^ aluminum silicate nanoparticles) E (+0.1 mL/L YSE in drinking water)	0.1 mL/L drinking water	27 d (from 15 to 42 days of age)	YSE reduces indoor ammonia concentration and alleviates visceral pathological damage; Decreases vaccine antibody levels; Elevates blood urea nitrogen and creatinine levels	Single dosage tested; Active composition and concentration of YSE undefined; Growth performance not evaluated	[103]
Broilers chicks (Ross 308)	Six groups with 300 broilers: T1 (Negative control, no antibiotics) T2 (Positive control + 100 mg/kg Terramycin) T3–T6 (+25, 50, 75, 100 mg/kg YSE, respectively)	25, 50, 75, 100 mg/kg diet (saponin ≥ 60%)	42 d	100 mg/kg YSE is identified as the optimal dosage; Enhances body weight gain, feed efficiency and nutrient digestibility; Improves intestinal morphology, carcass traits and meat quality	Only one broiler strain tested; Immune and antioxidant indices not measured; Higher dosages not evaluated	[109]
Broiler chicks (Cobb 500)	Three groups with 270 broilers: G1 (basal diet) G2 (+0.5 mL/L YSE in drinking water, 8 h/d) G3 (+1 mL/L YSE in drinking water, 8 h/d)	0.5 and 1 mL/L drinking water (Yucca Plus liquid^®^ (Beijing Xiqin Pharmaceutical Co., Ltd., Beijing, China) administered 8 h per day)	35 d	YSE reduces litter nitrogen content, total bacterial count and *Escherichia coli* abundance; Enhances systemic antioxidant capacity; Optimizes feed conversion ratio and protein utilization efficiency; Causes no hepatic or renal damage	Higher dosages and alternative administration routes not evaluated; Ammonia concentration not directly measured	[98]
Broiler chicks (Ranger Classic, initial BW: 33.5 ± 0.50 g)	Four groups with 140 broilers: C CEO (+1 mL/L of 5% of *C. aurantium* L. peel EO) Ys (0.05 mL/L of yucca saponins pure Extract) CEO + Ys	0.05 mL/L drinking water	63 d	Both CEO and Ys reduce oocyst shedding, alleviate intestinal damage and improve body weight gain; Ys elevates serum total protein and cholesterol levels; Neither treatment mitigates intestinal tissue inflammation	Single dosage tested; Field trial conditions are uncontrollable; No vaccine or antibiotic positive control	[110]
Broiler chicks (Cobb 500)	Four groups with 320 broilers: G1 (basal diet) G2 (+0.025 mL/L YSE in drinking water) G3 (+0.05 mL/L YSE in drinking water) G4 (+0.075 mL/L YSE in drinking water)	0.025, 0.05, 0.075 mL/L drinking water (DK YUCCA^®^)	35 d	0.075 mL/L YSE yields the best efficacy; Promotes growth and optimizes feed conversion ratio; Reduces litter nitrogen and moisture content; Enhances nutrient digestibility, antibacterial activity and antioxidant capacity	Direct measurement of ammonia emission not performed; No antibiotic control group included	[99]
Laying hens (Lohmann Pink, initial BW: 1.68 ± 0.03 kg, 58 weeks of age)	Three groups with 60 hens: CK YE (+0.1% YSE) AE (+0.1% astragalus extract)	0.1% YSE (equivalent to 1000 mg/kg diet)	21 d	YE inhibits key enzymes in nitrogen metabolism and elevates secretory IgA level; Enriches ammonia-suppressing intestinal microbiota; Does not affect laying performance; Exhibits weaker ammonia-reduction efficacy and economic benefit than astragalus extract	Single dosage tested; Short trial duration (21 d); Long-term effects on production performance not evaluated; Dosage consistency between in vitro and in vivo requires further validation	[105]
Laying hens (Hy-Line Brown, 27 weeks of age)	Two groups with 180 hens: CN (basal diet) MCC (+3340 mg/kg yucca extract compound preparation)	3340 mg/kg diet	12 weeks	Increases laying rate; Modulates serum immune indices; Optimizes the abundance of beneficial and pathogenic bacteria in feces	Single dosage tested; Active components of the compound preparation unknown; Egg quality indices not measured	[78]
Laying hens (Lohmann Gray, 35 weeks of age)	2 × 2 factorial design with 48 hens; main factors: YSE supplementation (0 vs. 500 mg/kg) and immune challenge (d 36–45)	500 mg/kg diet (30% active ingredients)	45 d (immune challenge from d 36 to d 45)	YSE activates the Nrf2 signaling pathway; Enhances intestinal antioxidant capacity and barrier function in laying hens; Alleviates stress-induced intestinal damage	Single dosage tested; Short challenge period; Long-term effects remain unknown	[100]
Laying breeder hens (White Plymouth Rock × Rhode Island Red cross, 27 weeks of age)	Four groups with 84 hens: CON V (+16.5 mg/kg virginiamycin) QY (+250 mg/kg Quillaja and Yucca) V + QY	250 mg/kg composite (Quillaja-dominant, Yucca-supplementary; ~4% total saponins)	From 30 to 49 weeks of age (5 cycles, 28 d per cycle)	Composite product optimizes feed conversion ratio and eggshell quality; Reduces cracked and soiled egg incidence; Outperforms virginiamycin in overall efficacy	Independent efficacy of yucca cannot be distinguished from the composite formulation; No single yucca control group; Mixed genetic background increases experimental variation	[97]

**Table 4:** Application effects of YSE supplementation in ruminants.

Animal Species	Experimental Design	Supplemental Dosage	Duration	Main Experimental Outcomes	Study Limitations	Refs
Holstein calves (100 days of age, initial BW: 90 ± 3.2 kg)	Two groups with 24 calves: G1 (basal diet) G2 (+0.555 g/head/d YSE)	0.555 g/head/d (De-Odorase^®^, Alltech Serdán, Mexico)	60 d	YSE alters ruminal fermentation and increases acetate concentration in calves; Induces systemic inflammation and oxidative stress; Aggravates hepatic load and reduces feed utilization efficiency	Single dosage tested; Fecal and urinary odor emissions not measured; Target organs responsible for inflammatory response not elucidated	[119]
Angus × Hereford calves (high weaning stress, initial BW: 220 ± 2 kg)	Three groups with 105 calves: CON (basal diet) M1 (+1 g/head/d YSE) M2 (+2 g/head/d YSE)	1 or 2 g/head/d (Micro-Aid^®^, DPI Global, Porterville, CA, USA) 18% saponins)	60 d	YSE improves body weight gain and feed efficiency; Reduces the treatment demand for bovine respiratory disease (BRD)	Ruminal fermentation parameters not measured; Methane emission not quantified;	[111]
Angus crossbred heifers (rumen-cannulated, initial BW: 224 ± 4 kg)	4 × 4 Latin square design, four groups with 16 heifers: CON (basal diet) YS1 (+1 g/head/d YSE) YS2 (+2 g/head/d YSE) YS4 (+4 g/head/d YSE)	1, 2 or 4 g/head/d (Micro-Aid^®^, 18% saponins)	4 periods × 28 d per period	YSE optimizes ruminal fermentation and improves growth performance; Fails to alleviate high-concentrate diet-induced ruminal frothy bloat; Exerts no anti-bloat effect	Bloat incidence not reduced (core hypothesis unconfirmed); Ruminal protozoa count not decreased (contrary to expectations); Methane emission not measured	[112]
Holstein calves (4 days of age, female, initial BW: 40 ± 5 kg)	Four groups with 40 calves: Y0 (+0 g//head/d YSE) Y3 (+3 g//head/d YSE) Y6 (+6 g//head/d YSE) Y9 (+9 g//head/d YSE)	3, 6 or 9 g/head/d YS	60 d	Daily YS supplementation enhances immunity and antioxidant capacity in pre-weaning calves; Improves feed efficiency and reduces diarrhea incidence; Shows antibiotic alternative potential, with 9 g/head/d as the optimal dosage	Ruminal fermentation not determined; Post-weaning performance not tracked	[114]
Angus steers (initial BW: 510.54 ± 41.27 kg)	Three groups with 45 steers: CON (basal diet) CAP (+1.5 g/head/d capsaicin) YSE (+2.4 g/head/d YSE)	2.4 g/head/d YSE (10% saponins)	90 d	YSE regulates ruminal fermentation and microbiota in beef cattle; Increases ammonia nitrogen, total volatile fatty acids and Bacteroidota abundance; Reduces ruminal pH, acetate proportion and Firmicutes abundance; Does not affect dry matter intake	Only capsaicin set as control, with no direct comparison of production performance between sole YSE and blank control; Methane emission not measured; Protozoa population not quantified	[113]
Angus crossbred beef steers (initial BW: 315 ± 3 kg)	2 × 2 factorial design, four groups with 120 steers: With or without monensin + tylosin (0.36 + 0.09 g/head/d) With or without YSE (4 g/head/d)	4 g/head/d YSE (Micro-Aid^®^, 18% saponins)	155 d	YSE optimizes ruminal environment with fermentation regulatory effects comparable to monensin; Exerts no growth-promoting effect; Is difficult to replace antibiotic growth promoters	Rumen fluid sampled from cannulated cows rather than experimental steers; Methane emission not detected	[127]
Lactating Holstein dairy cows (rumen-cannulated)	3 × 3 Latin square design, three groups with 6 cows: C (basal total mixed ration, TMR) D (+5 g/head/d YSE) DY (YSE + *Saccharomyces cerevisiae*)	5 g/head/d YSE (De-Odorase^®^Alltech^®^)	3 periods × 49 d per period	YSE alone or in combination remodels ruminal microbiota and reduces methanogenic archaea abundance; Does not improve lactation performance or nitrogen utilization efficiency; Decreases dry matter intake	Methane production not quantified	[118]
Lactating crossbred Holstein-Friesian dairy cows	Four groups with 20 cows: CON (TMR) PRO (+25 g/head/d *S. cerevisiae*) YS (+20 g/head/d YS) SW (+100 g/head/d dry seaweed)	20 g/head/d YS (105 g/kg saponins)	4 months	YS inhibits protozoa and reduces ruminal ammonia nitrogen and in vitro methane production; Does not improve lactation performance; Probiotics and seaweed show superior lactation-promoting effects	Dosage of seaweed group is much higher than YS group, resulting in ununified comparison basis	[123]
Jersey dairy cows (mid-lactation, 165 DIM)	Two groups with 18 cows: CON (basal diet) TRT (+ 5 g yeast + 3.5 g plant bio-choline + 2.8 g enzyme preparation + 2.5 g YSE per head per day)	2.5 g/head/d YSE (De-Odorase^®^ (Alltech, Nicholasville, KY, USA), 8–12% saponins, 3–5% polyphenols)	56 d	Composite additive inhibits ruminal protozoa and increases propionate concentration; Enriches xylanolytic bacteria and exerts antioxidant and anti-inflammatory effects; Synergistically improves milk production performance and feed utilization efficiency	Single dosage tested; Functional contribution of each component cannot be identified in composite formulation; Methane emission not measured	[116]
Awassi male lambs (3–4 months of age, initial BW: 26.5 ± 2.0 kg)	Three groups with 60 cows: CON (TMR) YS300 (0.3 g/kg DM YSE) YS600 (0.6 g/kg DM YSE)	0.3, 0.6 g/kg DM YSE	84 d	0.3 g/kg YSE improves growth performance; 0.6 g/kg YSE reduces fecal and urinary odor but impairs growth and carcass traits	Methane mitigation effect of each dosage not determined; Long-term feeding data beyond 84 days are lacking; Optimal supplementation dosage not clearly defined	[115]
Merino ewes (4–6 years of age, initial BW: 54.23 ± 2.93 kg)	Two groups with 15 ewes: CON (7 ewes fed basal diet) Y (8 ewes fed basal diet + 1.5 g/head/d YS) Additional in vitro ovarian tissue culture assay was performed	1.5 g/head/d YS	30 d	YS inhibits small follicle development in ewes; Promotes granulosa cell apoptosis and downregulates sex hormone levels; Alters ovarian follicle-stimulating hormone response and exerts side effects on ovine reproduction; This effect shows species differences, with positive effects observed in rabbits and pigs	Only single dosage tested; Actual reproductive indices such as ovulation rate and conception rate not evaluated	[124]

**Table 5:** Application effects of YSE supplementation in aquatic animals.

Animal Species	Experimental Design	Supplemental Dosage	Duration	Main Experimental Outcomes	Study Limitations	Refs
Nile tilapia (*Oreochromis niloticus*) (initial BW: 13.54 ± 0.32 g)	Four groups with 120 fish: CON (basal diet) Y (+ 0.2 g/kg YSE) B (+ 1.0 g/kg Bacillus subtilis) YB (+0.2 g/kg YSE + 1.0 g/kg *B. subtilis*)	0.2 g/kg diet YSE	65 d	Sole YSE supplementation reduces waterborne ammonia nitrogen and enhances immune and antioxidant capacities in tilapia; Exerts no growth-promoting effect; Combined application synergistically improves water quality and elevates growth performance and immune-antioxidant competence	Single dosage tested; Intestinal microbiota not analyzed	[128]
Nile tilapia (initial BW: 3.8 ± 0.05 g)	Six groups with 360 fish: YMS0 YMS0.1 (+0.1% YSE) YMS0.3 (+0.3% YSE) YMS0.5 (+0.5% YSE) YMS1.0 (+1% YSE) YMS2.0 (+2% YSE)	0.1%, 0.3%, 0.5%, 1%, 2% diet YSE (De-Odorase^®^ (Alltech Korea, SeochoGu, Seoul, Republic of Korea) 30% extract, 2.39% saponins)	10 weeks	Optimal dietary inclusion of 0.1–0.14% YSE (23.9–33.4 mg/kg saponin) promotes growth and enhances immunity and disease resistance	Water quality effects not evaluated; Elevated hepatic enzymes at high dosages require further safety validation	[134]
Nile tilapia (initial BW: 40 ± 5 g)	Five groups with 225 fish: Con (no ammonia exposure) A (ammonia exposure) BS+A (+0.012 g/m^3^ probiotic mixture BS + ammonia) YSE+A (+0.11 mL/m^3^ YSE + ammonia) BS + YSE + A (0.012 g/m^3^ BS + 0.11 mL/m^3^ YSE + ammonia)	0.11 mL/m^3^ water YSE (Sanolife AFM^®^-INVE Aquaculture, Belgium)	2-week pre-treatment + 72 h acute ammonia exposure (TAN 5 mg/L)	Combined BS and YSE yields the most prominent ameliorative effect against ammonia toxicity; Is recommended for ammonia toxicity control in aquaculture	Different ammonia concentrations not evaluated; Long-term growth effects not assessed	[130]
Nile tilapia (initial BW: 4.00 ± 0.05 g)	Five groups with 300 fish: Control group Ammonia exposure group (5 mg/L NH3) Ammonia + 6 mg/L YSE Ammonia + 8 mg/L YSE Ammonia + 10 mg/L YSE (administered every two days)	6, 8, 10 mg/L water YSE (3% saponins), administered every 2 days	3 months	YSE effectively alleviates ammonia poisoning via improving water quality and enhancing antioxidant and immune functions; 10 mg/L shows the best efficacy	Only waterborne administration route tested	[131]
Nile tilapia (initial BW: 42.22 ± 1.25 g)	Four groups with 120 fish: Control group YSE (+ 8 mg/L YSE) Ammonia exposure group YSE + ammonia exposure group	8 mg/L water YSE (3% saponins), administered every 2 days	4 weeks	YSE mitigates chronic ammonia poisoning through antioxidant, anti-inflammatory effects and regulation of energy metabolism and growth-related gene expression	Only one dosage evaluated; No challenge test performed; Short trial duration	[140]
Nile tilapia (initial BW: 14.69 ± 0.09 g)	Four groups with 240 fish: Control group (28 °C) Yucca (28 °C, +0.1% YSE) HS group (36 °C, heat stress) Yucca+HS (36 °C, +0.1% YSE)	0.1% diet YSE (Biopowder^®^ (Gentech Industries Group, Shanghai, China), 11.08% saponins)	2 weeks	0.1% YSE alleviates heat stress-induced intestinal injury, oxidative stress, endoplasmic reticulum stress and inflammation; Improves protein homeostasis via activating Nrf2 pathway and Hsp70	Only one dosage tested; Short experimental period; Long-term effects not assessed	[137]
Nile tilapia broodstock (female: 132.98–183.45 g; male: 175.61–211.28 g) and larvae	Four groups with 72 broodstock: Control Probiotic (+2 g/tank/week Bacillus mixture) Yucca (+2 cm^3^/tank/week YSE) Probiotic + Yucca (+2 g/tank/week composite)	2 cm^3^/tank/week water YSE (approx. 0.19 mL/m^3^/d; AQUA-YUCCA^®^, 100% YSE); 2 g/tank/week composite (UNI ECOTREAT^®^, multi-strain bacteria + 80 mg yucca saponins)	60 d for broodstock; 45 d for larvae	Combined waterborne application synergistically improves water quality and enhances reproductive performance and larval growth	Single dosage tested; Intestinal microbiota not analyzed	[80]
Nile tilapia broodstock (female: 109.98 ± 1.48 g; male: 140.63 ± 1.62 g) and larvae	Four groups with 96 broodstock: CA (no water exchange) CB (50% weekly water exchange) Yucca (no water exchange + 0.5 mL/tank/day YSE) Biofloc (biofloc system)	0.5 mL/tank/day water YSE (approx. 0.33 mL/m^3^/d; AQUA-YUCCA^®^, 100% YSE)	84 d for broodstock; 45 d for larvae	Both biofloc system and YSE supplementation improve water quality and elevate reproductive efficiency of broodstock and larval quality; YSE can serve as a supplementary strategy for biofloc technology	Gonadal histomorphological changes not detected; Effects of yucca on sperm quality not evaluated; Yucca dosage not gradient-optimized; Long-term multi-generational reproductive effects not assessed	[142]
European seabass (initial BW: 5.0 ± 0.5 g)	Four groups with 180 fish: Control group YE0.25 (+0.25 g/kg YSE) YE0.5 (+0.5 g/kg YSE) YE1 (+1 g/kg YSE)	0.25, 0.5, 1 g/kg diet YSE (Vime-Yucca (P)^®^ (Vemedim Corporation, Vietnam), 33% YSE+12% saponins)	45 d	Dietary YSE improves water quality, promotes growth, enhances immunity and reduces farming cost; 1 g/kg is recommended	Short-term trial (45 d); Flesh quality not evaluated	[132]
European seabass (initial BW: 5.83 ± 0.02 g)	2 × 5 factorial design, 4500 fish: Stocking density (low, 100 fish/m^3^; high, 200 fish/m^3^) Water treatment (control, 10 g/L zeolite, 15 g/L zeolite, 0.75 mL/L YSE, 1 mL/L YSE)	0.75, 1 mL/L water YSE (Yucca100-AP^®^ (Aqualand Co. Tanta, Egypt), 500 g/L YSE + 300 g/L saponins + 100 g/L polyhydroxy stilbenes)	45 d	Both YSE and zeolite improve water quality and fish health under high-density culture; 15 g/L zeolite outperforms YSE	Only two YSE levels set; Antioxidant enzymes not detected	[135]
European seabass (initial BW: 5.0 ± 0.5 g)	Four groups with 180 fish: Y0 Y1 (+0.25 mL/L YSE) Y2 (+0.5 mL/L YSE) Y3 (+0.75 mL/L YSE)	0.25, 0.5, 0.75 mL/L water YSE	45 d	Waterborne YSE effectively reduces ammonia nitrogen, improves survival rate and enhances immunity and growth; 0.75 mL/L is recommended	Antioxidant indices not detected; No challenge test performed; Different administration frequencies not evaluated	[136]
Japanese eel (initial BW: 9 ± 0.2 g)	Nine groups with 450 fish: FMA0 FMA10, 20, 30, 40 FMA0 + 0.1% YSE FMA0 + 0.4% SG FMA20 + 0.1% YSE FMA20 + 0.4% SG	0.1% diet YSE (De-Odorase^®^, 30% yucca extract, 2.39% saponins)	8 weeks	0.1% YSE supplementation elevates the optimal FMA replacement ratio of fish meal from 10% to 20% without compromising growth and immunity	Only one YM dosage set; Sole YM effect not independently evaluated	[133]
Zebrafish (3–4 months of age, 3–4 cm body length)	10 fish per group (1 h/d, 8 days): Control group Scopolamine group (Sco, 100 μmol/L) Sco + YS pur (1, 3, 5 μg/L) Sco + YS poly (1, 3, 5 μg/L) Sco + YuB (1, 3, 5 μg/L) Sco + GloA (1, 3, 5 μg/L)	1, 3, 5 μg/L water YS pur (purified phenolics, 50.3% spiro-flavonostilbenes), YS poly (polymerized fraction), YuB, GloA	8 d	Yucca phenolics and spiro-flavonostilbenes exert neuroprotective effects via antioxidant and anti-inflammatory mechanisms	Blood–brain barrier penetration of compounds not verified; Intestinal microbiota metabolites not analyzed	[141]
Rainbow trout (initial BW: 10.3 ± 0.1 g)	Four groups with 240 fish: CO (basal diet) GPS 1 (+1.35 g/kg composite) GPS 2 (+2.025 g/kg composite) GPS 3 (+2.7 g/kg composite)	1.35, 2.025, 2.7 g/kg diet composite (Biorigin^®^ (Sao Paulo, Brazil), containing β-glucan, proanthocyanidins and saponins)	90 d	GPS 2 (2.025 g/kg) delivers the most balanced benefits in growth, immunity, intestinal health and water quality improvement	Independent effects of each component not evaluated	[129]
Pacific white shrimp (*Litopenaeus vannamei*) (initial BW: 0.03–0.05 g)	Twelve concrete tanks (8 m × 8 m × 1.3 m), stocked at 500 PL/m^2^, four groups: OY-0 (no product; weekly 30–40% water exchange. OY-1 (+0.20 g/m^2^/week of oak–yucca composite product, OY). OY-2 (+0.25 g/m^2^/week OY). OY-3 (+0.3 g/m^2^/week OY).	0.20, 0.25, 0.30 g/m^2^/week water OY (Sapotan Powder^TM^ (OY, Tanin Sevnica, Slovenia))	90 d	Reduce water ammonia nitrogen and improve water quality; Lower FCR and enhance growth performance; No significant effects on shrimp body composition	Mechanism remains unclear; Experimental duration was short	[138]
Pacific white shrimp (*Penaeus vannamei*) (PL_12_, initial BW: ~0.003 g)	Twenty-one 100 L glass tanks, stocked at 100 shrimp/tank, seven groups: C (Control) Y (+1 mg/L YSE). ABB (+5 × 10^4^ CFU/mL Bacillus consortium). AY (+5 × 10^8^ CFU /mL Bacillus AQ_1_ + 1 mg/L YSE) B2Y (+5 × 10^8^ CFU /mL Bacillus BIO_2_ + 1 mg/L YSE) B3Y (+5 × 10^8^ CFU /mL Bacillus BAL_3_ + 1 mg/L YSE) ABBY (+5 × 10^8^ CFU /mL Bacillus AQ_1_, BIO_2_, BAL_3_ +1 mg/L YSE)	1 mg/L/week water YSE	35 d	Reduce ammonia nitrogen and inhibit pathogenic bacteria; Improve growth performance and lower FCR	Trial duration was short; Synergistic mechanism remains unelucidated	[139]

**Table 6:** Cross-species comparison of YSE application effects.

Animal Category	Optimal Supplementation Dosage	Main Functional Effects	Proposed Mechanisms of Action	Study Limitations	Refs
Weaned piglets	120 mg/kg diet	Improves growth performance; Ameliorates intestinal health; Reduces nitrogen emission; Enhances immune function; Improves antioxidant capacity	Upregulates gene expression of tight junction proteins and mucins; Increases digestive enzyme activities and nutrient transporter expression; Enriches beneficial bacteria and suppresses pathogenic bacteria; Downregulates pro-inflammatory cytokine expression; Activates the Nrf2 antioxidant pathway to scavenge free radicals	Lack of discussion regarding the toxicity threshold at high doses; Synergistic mechanisms with enzymes, probiotics or zinc remain unclear; The trial duration is short, and there is a lack of long-term effect evaluation.	[39,77,86,89,91]
Finishing pigs	125–300 mg/kg diet	Improves growth performance; Reduces harmful gas emission; Modulates nitrogen metabolism; Improves meat quality	Reduces ammonia via inhibiting urease activity or direct ammonia binding; Regulates metabolism by altering circulating levels of protein catabolites	Limited recent studies available; Optimal dosage remains debatable; Growth-promoting effects are inconsistent; Sex-dependent response differences are unclear	[90,91,93]
Sows	Replacement gilts: 250 mg/kg diet; Gestating and lactating sows: 600 mg/kg diet	Improves reproductive performance; Reduces diarrhea incidence in piglets; Enhances nutrient digestibility; Ameliorates antioxidant status; Reduces fecal nitrogen loss; Modulates intestinal microbiota	Binds ammonia or suppresses ammonia production; Scavenges free radicals; Saponins exert estrogen-like effects to promote oestrus and ovarian function recovery; Enriches beneficial bacteria and suppresses pathogenic bacteria	Limited recent studies available; Optimal dosage remains debatable; Long-term studies on growth performance of offspring from gestating and lactating sows are lacking	[87,88]
Broilers	Growth promotion: 100–125 mg/kg diet; Anticoccidial & intestinal protection: 500 mg/kg diet; Drinking water administration: 0.5–1 mL/L (8 h/d)	Increases body weight gain and reduces FCR; Decreases litter nitrogen content and ammonia emission; Modulates intestinal microbiota; Exerts anti-inflammatory and antioxidant effects; Exhibits anticoccidial activity	Saponins bind to cholesterol in parasite cell membranes and disrupt membrane integrity (anticoccidial effect); Surfactant properties of saponins promote fat emulsification and nutrient absorption; Polyphenols scavenge free radicals and activate the Nrf2 pathway to enhance antioxidant defense; Reduces intestinal urease and uricase activities and binds ammonia to decrease nitrogen emission; Enriches beneficial bacteria and suppresses pathogenic bacteria; Improves intestinal villus height-to-crypt depth ratio and enlarges absorptive surface area	Most studies adopt single dosage without dose–response curves; Large compositional variations among yucca whole powder, saponin extracts and composite products hinder cross-study comparison; Circulating immune indices (cytokines, antioxidant enzymes) are mostly undetermined or partially measured; Studies on long-term feeding (>42 d) and effects on meat flavor are insufficient	[83,94,95,96,98,99,101,102,103,104,106,107,109,110]
Laying hens	100–500 mg/kg diet	Increases laying rate; Improves eggshell strength, thickness and cuticle quality; Reduces defective and soiled egg rates; Decreases excreta moisture and ammonia emission; Exerts anti-inflammatory effects; Modulates intestinal microbiota; Elevates hatchability and reduces early embryonic mortality	Saponins bind ammonia to reduce intestinal and indoor ammonia concentrations; Promotes calcium absorption and shell gland secretion to enhance eggshell ultrastructure; Polyphenols exert anti-inflammatory and antioxidant effects to protect oviduct and ovarian function; Enriches beneficial bacteria and suppresses pathogenic bacteria; Upregulates tight junction proteins ZO-1 and occludin; Elevates serum T3 and T4 levels to improve energy metabolism and laying performance	Far fewer studies on laying/breeder hens than on broilers; Most are short-term trials (≤19 weeks) lacking data across the full laying cycle (up to 72 weeks); Independent contribution of yucca cannot be isolated from synbiotic-yucca composite products; Systematic evaluation of effects on offspring chick health is lacking	[78,97,100]
Calves	Pre-weaning: 9 g/head/d; Growing period: 0.5–4 g/head/d (use with caution)	Beneficial effects: Optimizes ruminal fermentation in calves; Improves growth and feed efficiency; Reduces treatment demand for respiratory diseases; Enhances immune and antioxidant capacities; Reduces diarrhea incidence; Adverse effects: Increases ruminal acetate concentration; Induces systemic inflammation and oxidative stress; Aggravates hepatic load; Fails to alleviate high-concentrate-induced ruminal frothy bloat	Beneficial: Saponins inhibit intestinal pathogenic bacteria; Polyphenols/resveratrol scavenge free radicals and enhance antioxidant enzyme activities; Adverse: High-dose saponins induce local intestinal inflammation via membrane lysis, leading to LPS translocation and systemic endotoxaemia; Energy is reallocated from growth to immune defense; Saponin deglycosylation in fiber-rich diets causes accumulation of toxic sapogenins	Limited recent dosage studies; Fecal and urinary odor emission not measured; Post-weaning effects not followed up	[111,112,114,119]
Beef cattle	2.4–4 g/head/d	Regulates ruminal fermentation and microbiota in beef cattle	Saponins exert immunoadjuvant effects to enhance antigen presentation and lymphocyte response; Inhibits *S. bovis* and *B. fibrisolvens* and promotes propionate-producing bacteria to enhance gluconeogenesis; Suppresses lactate-producing bacteria to stabilize ruminal pH; Enriches beneficial bacteria and suppresses pathogenic bacteria	Limited recent dosage studies; Lack of dose–response investigation experiments	[113,127]
Dairy cows	Recommended for combined application	Alone or in combination remodels ruminal microbiota; Reduces methanogenic archaea abundance; Decreases ammonia nitrogen and in vitro methane production; Yucca-containing composite additives synergistically improve milk production performance and feed utilization efficiency	Saponins in composite additives inhibit protozoa and enhance gluconeogenesis; Polyphenols exert antioxidant effects to reduce lipid peroxidation; Saponins suppress partial *Prevotella* spp. to alter microbiota balance in protein and carbohydrate metabolism; Reduced methanogenic archaea may decrease hydrogen utilization efficiency	Composite additive design prevents isolation of independent yucca contribution; There are significant variations in the dosage of YSE, and there is a lack of research on dose gradients.	[116,118,123]
Sheep	Not recommended	Beneficial: Reduces ruminal ammonia nitrogen concentration and odorous gas emission; Adverse: Approximately 0.89 g/head/d reduces average daily gain and feed efficiency in lambs and impairs partial carcass traits; Impairs reproductive performance in ewes	Beneficial: Selectively inhibits protozoa to reduce ammonia production and blood urea nitrogen; Adverse: High-dose saponins alter intestinal permeability; Steroidal saponins act as phytestrogens to competitively bind estrogen receptors and inhibit endogenous E2 synthesis; Reduces serum cholesterol and decreases P4 synthesis substrates; Directly induces granulosa cell apoptosis	Limited recent relevant studies; Long-term effects not evaluated	[115,124]
Aquatic animals (dietary administration)	1–2 g/kg diet, or combined with probiotics and enzymes	Indirectly reduces waterborne ammonia nitrogen; Promotes growth and thus feed conversion efficiency; Ameliorates intestinal health; Exerts anti-inflammatory and antioxidant effects; Enhances non-specific immunity; Alleviates stress; Improves post-challenge survival rate	Steroidal saponins and carbohydrate fractions bind ammonia; Saponins alter intestinal epithelial membrane structure to reduce surface tension and promote nutrient absorption; Polyphenols activate the Nrf2-ARE pathway to enhance antioxidant enzymes; Inhibits NF-κB to alleviate inflammation; Suppresses UPR to mitigate endoplasmic reticulum stress; Non-covalent complexation of polyphenols and β-glucan enhances stability; Saponins reduce blood ammonia to decrease protein catabolism	Safety of high dosage (10 g/kg) requires further validation; Effects of long-term feeding (>3 months) on intestinal microbiota colonization and flesh quality remain unknown; Mechanisms underlying species-specific sensitivity to yucca are unclear; Optimal ratio of components in combined supplementation not systematically optimized	[129,132,133,134,137]
Aquatic animals (waterborne administration)	Solid form: 8–10 mg/L water; Liquid form: 0.75–1 mL/L water	Significantly reduces total ammonia nitrogen in water; Elevates dissolved oxygen level; Indirectly improves growth and feed conversion ratio; Exerts anti-inflammatory and antioxidant effects; Promotes reproduction; Alleviates hepatic and renal damage	Steroidal saponins and carbohydrate fractions directly bind/chelate ammonia; Saponins lower water pH to reduce NH_3_/NH_4_^+^ ratio; Phenolics scavenge free radicals and activate the Nrf2-ARE pathway to enhance endogenous antioxidant enzymes; Inhibits the NF-κB pathway to alleviate inflammation; Reduces cortisol to mitigate stress	Water administration frequency (daily vs. every other day vs. every 72 h) lacks systematic optimization; Stability of yucca bioactive components in water (photolysis, microbial degradation) not evaluated; Effects of long-term application on aquaculture ecosystems (plankton, sediment microbiota) remain unknown	[80,130,131,135,136,140,141]

## Data Availability

No new data were created or analyzed in this study. Data sharing is not applicable to this article.

## Abbreviations

The following abbreviations are used in this manuscript:

YSE	Yucca schidigera extract
UAE	Ultrasound-assisted extraction
DES	Deep eutectic solvents
NMR	Nuclear magnetic resonance
LC-MS	Liquid chromatography-mass spectrometry
Nrf2	Nuclear factor erythroid 2-related factor 2
ARE	Antioxidant response element
SOD	Superoxide dismutase
GSH-Px	Glutathione peroxidase
CAT	Catalase
COX-1	Cyclooxygenase-1
COX-2	Cyclooxygenase-2
PGE2	Prostaglandin E2
NF-κB	Nuclear factor-kappa B
MAPK	Mitogen-activated protein kinase
TNF-α	Tumor necrosis factor-α
IL-6	Interleukin-6
Keap1	Kelch-like ECH-associated protein 1
HO-1	Heme oxygenase-1
ROS	Reactive oxygen species
ERK1/2	Extracellular signal-regulated kinase 1 and 2
JNK	c-Jun N-terminal kinase
JAK2	Janus kinase 2
STAT3	Signal transducer and activator of transcription 3
O-GlcNAc	O-linked N-acetylglucosamine
mRNA	Messenger ribonucleic acid
T-AOC	Total antioxidant capacity
MDA	Malondialdehyde
SCFAs	Short-chain fatty acids
4-HPAA	4-Hydroxyphenylacetic acid
V/C	Villus height-to-crypt depth ratio
ADG	Average daily gain
ADFI	Average daily feed intake
FCR	Feed conversion ratio
G:F	Gain-to-feed ratio
FI	Feed intake
OPG	Oocyst per gram
DMI	Dry matter intake
VFA	Volatile fatty acid
NH3-N	Ammonia nitrogen
TVFA	Total volatile fatty acid
DM	Dry matter
CP	Crude protein
NDF	Neutral detergent fiber
OM	Organic matter
TMR	Total mixed ration
DIM	Days in milk
LCA	Life-cycle assessment
GRAS	Generally recognized as safe
FDA	Food and Drug Administration
EFSA	European Food Safety Authority
EC	European Community
TAN	Total ammonia nitrogen
2D-NMR	Two-dimensional nuclear magnetic resonance
HPLC-ELSD	High-performance liquid chromatography with evaporative light scattering detection
UV	Ultraviolet
YPB	Yucca schidigera polysaccharide B
Rha	Rhamnose
Araf	Arabinofuranose
Galp	Galactopyranose
Glcp	Glucopyranose
MR	Mannose receptor
EGFR	Epidermal growth factor receptor
LPS	Lipopolysaccharide
Hsp70	Heat shock protein 70
UPR	Unfolded protein response
TBARS	Thiobarbituric acid reactive substances
BRD	Bovine respiratory disease
AZn	Amino-zinc
CNKI	China National Knowledge Infrastructure
MUC-2	Mucin-2
ZO-1	Zonula occludens-1
IgA	Immunoglobulin A
IgM	Immunoglobulin M
IgG	Immunoglobulin G
IgY	Immunoglobulin Y
IFN-γ	Interferon-γ
IL-1β	Interleukin-1β
IL-2	Interleukin-2
IL-8	Interleukin-8
IL-10	Interleukin-10
PI3K	Phosphoinositide 3-kinase
AKT	Protein kinase B
MAP	Mitogen-activated protein
CAMK2G	Calcium/calmodulin-dependent protein kinase II gamma
MKNK1	MAP kinase-interacting serine/threonine kinase 1
16S rRNA	16S ribosomal RNA
BWG	Body weight gain
F/G	Feed-to-gain ratio
EE	Ether extract
TAC	Total antioxidant capacity
